# Metagenomic analysis and functional characterization of the biogas microbiome using high throughput shotgun sequencing and a novel binning strategy

**DOI:** 10.1186/s13068-016-0441-1

**Published:** 2016-02-02

**Authors:** Stefano Campanaro, Laura Treu, Panagiotis G. Kougias, Davide De Francisci, Giorgio Valle, Irini Angelidaki

**Affiliations:** Department of Biology, University of Padua, Via U. Bassi 58/b, 35131 Padua, Italy; Department of Environmental Engineering, Technical University of Denmark, 2800 Kongens Lyngby, Denmark

**Keywords:** Anaerobic digestion, Biogas, Metagenomics, Next-generation sequencing, Microbial community structure, Binning, Methanogens, Archaea, Bacteria

## Abstract

**Background:**

Biogas production is an economically attractive technology that has gained momentum worldwide over the past years. Biogas is produced by a biologically mediated process, widely known as “anaerobic digestion.” This process is performed by a specialized and complex microbial community, in which different members have distinct roles in the establishment of a collective organization. Deciphering the complex microbial community engaged in this process is interesting both for unraveling the network of bacterial interactions and for applicability potential to the derived knowledge.

**Results:**

In this study, we dissect the bioma involved in anaerobic digestion by means of high throughput Illumina sequencing (~51 gigabases of sequence data), disclosing nearly one million genes and extracting 106 microbial genomes by a novel strategy combining two binning processes. Microbial phylogeny and putative taxonomy performed using >400 proteins revealed that the biogas community is a trove of new species. A new approach based on functional properties as per network representation was developed to assign roles to the microbial species. The organization of the anaerobic digestion microbiome is resembled by a funnel concept, in which the microbial consortium presents a progressive functional specialization while reaching the final step of the process (i.e., methanogenesis). Key microbial genomes encoding enzymes involved in specific metabolic pathways, such as carbohydrates utilization, fatty acids degradation, amino acids fermentation, and syntrophic acetate oxidation, were identified. Additionally, the analysis identified a new uncultured archaeon that was putatively related to *Methanomassiliicoccales* but surprisingly having a methylotrophic methanogenic pathway.

**Conclusion:**

This study is a pioneer research on the phylogenetic and functional characterization of the microbial community populating biogas reactors. By applying for the first time high-throughput sequencing and a novel binning strategy, the identified genes were anchored to single genomes providing a clear understanding of their metabolic pathways and highlighting their involvement in anaerobic digestion. The overall research established a reference catalog of biogas microbial genomes that will greatly simplify future genomic studies.

**Electronic supplementary material:**

The online version of this article (doi:10.1186/s13068-016-0441-1) contains supplementary material, which is available to authorized users.

## Background

Biogas production from agricultural and industrial wastes allows the simultaneous treatment of organic residues with generation of a versatile energy carrier (i.e., methane), which in turn can be transformed into electricity and heat [1]. The biogas is produced through a biologically mediated process, the so called “anaerobic digestion” (AD), which is divided into four steps, namely hydrolysis, acidogenesis, acetogenesis, and methanogenesis [2]. All steps are executed by an extremely specialized and complex microbial community, in which different members have distinct roles in a collective organization [3]. These intricate sets of relationships between the microorganisms hamper the investigation of the microbial community through traditional microbiological methods. To address this problem is mandatory to go beyond a simple identification of the microbial species, unveiling their functional roles in the biogas production system.

In most of the shotgun sequencing studies performed on the AD microbial community, the functional characterization of the microbes was performed directly on the short reads [4] without prior assembly, or at best, gene finding was performed on a small number of short scaffolds [5, 6]. However, it is well known that a high-quality assembly strongly improves the reliability of gene finding and annotation [7]. Only recently, the metagenome of a single biogas plant was assembled but it was not binned to extract genomes [8].

Till now, the identification of species in the biogas community was performed using sequence similarity search against reference genomes present in public databases [5, 9, 10]. However, these genomes were isolated from different environments and, even if they belong to phylogenetically related groups, they might have different functional properties. For this reason, a “predictive metagenomic approach,” based on 16S gene sequencing, was tentatively applied to the AD microbial community [3], but it was found that this strategy is more reliable when adequate reference genomes are available in the public databases [3, 11]. Another weak point of many metagenomic studies is that they are conducted without performing assembly and binning processes. It is known that short reads are error prone and contain only minimal signal for homology searches, hampering direct annotation against reference databases [12]. As a consequence, the AD microbiome still remains a “black box” due to the small number of complete microbial genomes obtained from the biogas community [13, 14]. Therefore, the development of an appropriate database representing the biogas microbial community will allow the correlation of genome characteristics, phylogenetic, and metabolic properties of these uncultivated microorganisms.

To achieve this outcome is mandatory to perform a *de novo* assembly of the shotgun sequence data. This process offers several advantages for analyzing metagenomics datasets, as for example: (a) improved accuracy of sequences obtained by removing random sequencing errors, (b) more reliable gene finding and annotation process, (c) significant reduction of the data for subsequent processing and obviously, (d) possibility to discover novel genomic elements. Despite these advantages, *de novo* assembly in microbial community is extremely complex because it results in a large set of scaffolds that are difficult to be classified in single biological entities [i.e., Genomes Bin (GB)]. This classification was previously performed on other microbial communities with different methods such as tetranucleotide composition assignment [15], tetranucleotide composition combined with abundance of the scaffold in a small number of conditions [16], and binning of co-abundant genes across a series of metagenomic samples [17]. These methods are based on the rationale that the relative concentrations of a microbial species can change in different contexts; thus, scaffolds can be assembled in the same individual genome if their coverage changes concertedly. This process is called binning. Nevertheless, procedures based on nucleotide genomic composition can be inconsistent due to uneven tetranucleotide distribution into the same genome. On the other hand, abundance-based methods cannot give comprehensive segregation of all entities in complex samples [16, 18] and a reliable binning of co-abundant genes requires numerous samples. Recently, fully automated binning procedures have been developed such as CONCOCT [19], GroopM [20], or MetaBAT [21].

In this study, we applied a novel two-stage approach strategy, combining the two procedures previously proposed [16, 17]. This allowed the extraction of 106 GBs from the biogas microbial community which adds a new chapter in the study of the anaerobic digestion (AD). Annotation of the identified genes and functional analysis of the species gave for the first time a clear understanding of the AD microbiome and allowed to establish a reference collection of biogas microbial genomes that will greatly simplify future genomic studies.

## Results and discussion

Approximately, 340 millions high-quality paired-end reads (~51 gigabases of metagenomic sequence) were obtained from high throughput sequencing of 15 samples collected from 8 anaerobic digesters, representing conventional biogas reactors. The assembly of the reads resulted in 409,831 scaffolds (~686 Mbp) ranging in size from 500 to 313,754 bp (N50 2338). The percentage of reads aligned to the assembly varied from 57 to 73 % (with a mean average of 67 %) as shown in Additional file 1: Table S1. No differences were found between the samples included in the assembly and those used only for the binning process, suggesting that the assembly was fairly representative for all the reactors. It should be mentioned that ~242 Mbp are in scaffolds larger than 5 kbp (“Methods” and Additional file 1). After the assembly process, the gene finding and annotation are more reliable and led to the identification of nearly one million protein encoding genes, 23.6 % of which could be assigned to GBs (Additional file 2). The protein encoding genes were annotated using COG [22], KEGG [23], and Pfam [24] (Additional file 2). The results showed that 569,645 genes (60.8 %) had a match in the COG database, 418,103 (44.6 %) in KEGG and 579,337 (61.8 %) had a protein domain annotated in Pfam. Finally, 277,604 genes (29.6 %) were completely unknown. The number of predicted proteins is approximately 70 times and 3.7 times more than those obtained in the two best previous assemblies of a biogas microbial community reported in the literature [6, 8].

The number of genes belonging to each KEGG category in the assembly was compared with the scaffold coverage, which is directly related to species abundance (Additional file 1 and Additional file 1: Figure S2). This means that the categories with the higher ratio between “coverage” and “number of genes in the category” are those associated with most abundant GBs. This analysis allowed an evaluation of the relevance of the KEGG classes considering both the number of genes in the pathway and the abundance of the species in the microbial community. From these data, it was evident that some metabolic pathways included genes with a high average coverage because they were encoded (also) in the genomes of the more abundant species of the microbial community as shown in Additional file 1: Figure S2. The metabolic pathway of methanogenesis is the most straightforward example indicating that some methanogenic archaea (i.e., Eu01) dominate the microbial community in terms of abundance. We can assume that, for the same reason, the riboflavin KEGG pathway, which led to the biosynthesis of the proteins’ cofactor F430 involved in methanogenesis [25], is one of the highly ranked in the list.

On the contrary, the KEGG pathway modules related to the degradation of xenobiotic compounds like “styrene,” “naphthalene,” “fluorobenzoate,” and “aromatic compounds” were mainly encoded in low abundant species and frequently belong to scaffolds that could not be assigned through the binning process. For example, only 22 % of the genes involved in “xylene degradation” were binned vs. 36 % of the “RNA-transport” and 33 % of the “riboflavin metabolism” (Additional file 2). This suggests that the degradation of xenobiotic compounds is specific to the rare biosphere in the biogas reactors. The only notable exception is “nitrotoluene degradation” but this is expected as the degradation of this compound and incorporation into the bacterial biomass in anaerobic conditions has been previously demonstrated [26–28].

Carbohydrate phosphotransferase (PTS) system, despite being represented by 1261 genes, is the second least abundant category considering the ratio “coverage/number of genes.” This suggests that mainly low abundant community members utilize this system to transport sugars. This result is totally unexpected, as PTS is widely spread among bacteria [29]. However, our data evidence that, in the AD microbiome, ABC transporters are more frequent in the high abundant species.

Moreover, it was found that “nitrogen metabolism” includes genes mostly represented in low abundance species. This could be due to the average low nitrogen concentration contained in cattle manure (in comparison for example to pig or poultry manure) [30]. It can be expected that, due to the high dynamicity of the biogas community [3, 14, 31], modification of the manure composition (for example a higher quantity of ammonia) can lead to an increase in the abundance of some species that in our experiment are associated to the rare biosphere.

### Binning process and taxonomic classification

Mapping reads from each sampling point to the assembled scaffolds indicated that the microbial species were differentially represented due to heterogeneous manure feedstock composition. The differences in the microbial abundance allowed the clustering of the scaffolds and resulted in the extraction of 106 GBs from the total assembly. A detailed explanation of the binning assembly procedure is reported in Additional file 1 together with a schematic representation of the binning strategy in Additional file 1: Figure S3. In the first part of our procedure, high-quality GBs were manually extracted using the procedure of Albertsen et al. [16]. These GBs served as internal controls and were used to drive the second part of the binning. By an automatic extraction process based on clustering of scaffolds having similar coverage profiles, 61 additional GBs were identified. The estimated completeness of the GBs, based on the presence of 107 conserved marker genes [32], ranged from 15 % to more than 99 % (with a mean of 83 %) (Additional file 3). In order to validate this finding, an additional analysis was performed using CheckM [33], which gave as output very similar values (85 % completeness on average). The level of genome contamination was estimated both considering the number of duplicated essential genes and also with CheckM; the contamination was found to be extremely low and ranging on average between 3 and 5 % depending on the method used (Additional file 3). With our procedure, we have successfully identified 60 genomes with estimated completeness higher than 90 % considering the 107 essential genes, or 51 genomes according to CheckM. The result obtained is of a very high quality if compared with previous studies obtained from single-cell genome sequencing, where genome completeness averages around 40 % [34].

An additional analysis was performed using MetaBAT software [21] in order to evaluate the performance of our binning strategy. Considering as thresholds a) genome completeness higher than 90 % and b) contamination level lower than 20 %, MetaBAT managed to extract 42 GBs, while our binning strategy led to the identification of 51 GBs. Even by lowering the completeness threshold (e.g., to 70 %), our binning strategy was able to extract more GBs. The outcome of this comparison validated the high accuracy and efficiency of the binning strategy presented in the current manuscript.

**Fig. 1:**
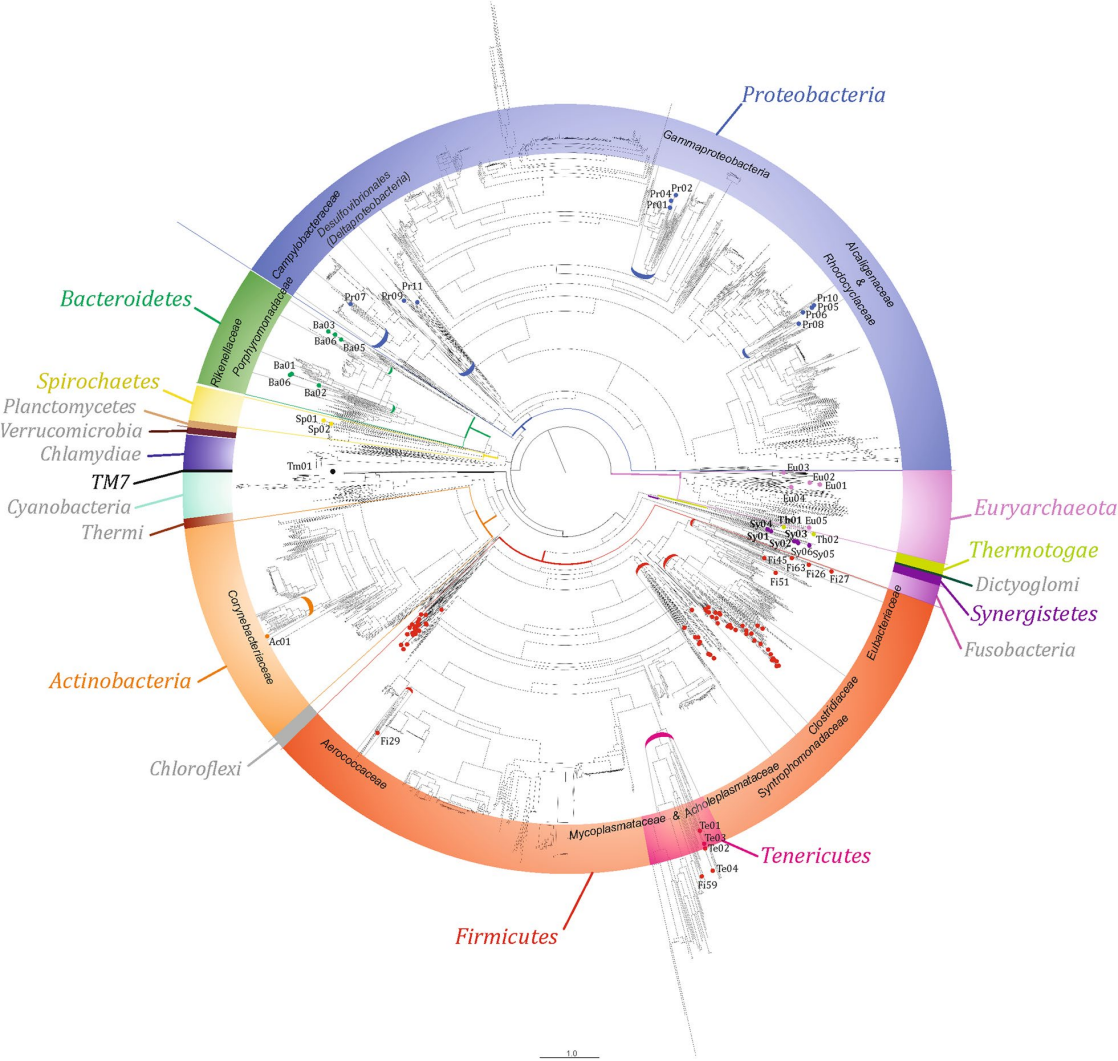
Phylogenetic assignment of the 106 GBs. High-resolution microbial tree of life with taxonomic annotations, microbial phylogeny, and putative taxonomy, obtained with PhyloPhlAn using 400 broadly conserved proteins used to extract phylogenetic signal [66]. The tree was built using FigTree and contains a total of 3737 microbial genomes plus the 106 GBs identified (represented by *small colored dots*). Organisms are *colored* based on phyla, those in *light grey* color *text*, were absent

Taxonomic assignment showed that none of the GBs could be assigned to species level, only 10 GBs were assigned to the genus level, while the vast majority were assigned to phylum level (Table 1; Additional file 3). This confirms that most of the species in the biogas microbial community were not previously characterized at a genomic level [35]. The more affordable taxonomic assignments were obtained for *Euryarchaeota* Eu01, Eu02, Eu04, Eu05 suggesting that archaea are better characterized than bacteria in the biogas community (with the exception of Eu03). On the contrary, bacteria are completely unknown at genomic level. The results revealed that the biogas microbial community is dominated by the phylum *Firmicutes* (69 GBs) followed by the phyla *Proteobacteria* (10 GBs) and *Bacteroidetes* (6 GBs), which is in accordance with other studies [36–40] (Fig. 1). Sixty-nine of the GBs belong to *Firmicutes* (Fig. 5; Additional file 3). The species included in this division are extremely relevant from a functional point of view since they are involved in many metabolic processes including the degradation of carbohydrates, fatty acids utilization, Wood–Ljungdahl pathway (WLP) (homoacetogenesis) or syntrophic acetate oxidation (SAO). The comprehensive high-resolution microbial tree (Fig. 1) evidenced that these GBs can be subdivided into six main sub-groups (Additional file 3). Three GBs belong to *Eubacteriaceae*, 17 to the family *Clostridiaceae*, seven to the family *Syntrophomonadaceae* and 22 can be assigned only to the class *Clostridia* and are distantly related to the other *Firmicutes*. It is worth mentioning that the GBs assigned to the class *Clostridia* are the most cryptic inside the community, as they are distantly related to other *Firmicutes*, showing deeply branched GBs (Fig. 1). Moreover, four of the GBs initially assigned to the *Firmicutes* using the 107 essential genes were then re-assigned to the family *Acholeplasmataceae*, of the phylum *Tenericutes* (Te01, Te02, Te03, Te04) using Phylophlan.

**Table 1:** Taxonomic assignment and basic genome characteristics of the 106 GBs extracted from biogas reactors

Genome bin ID	Genome bin “species name”	GB size (Mbp)	Estimated completeness (%)	Genome bin ID	Genome bin “species name”	GB size (Mbp)	Estimated completeness (%)
Pr02	*Gammaproteobacteria sp. DTU038*	4.2	84	Fi16	*Clostridia sp. DTU025*	2.0	95
Fi48	*Clostridiaceae sp. DTU079*	3.1	99	Fi13	*Clostridia sp. DTU022*	2.0	89
Fi49	*Clostridia sp. DTU080*	3.1	86	Fi32	*Clostridiales sp. DTU060*	2.0	88
Pr05	*Alcaligenaceae sp. DTU041*	2.9	96	Fi21	*Halothermothrix sp. DTU029*	2.0	94
Fi40	*Clostridiales sp. DTU070*	2.9	97	Ac01	*Actinomycetales sp. DTU046*	1.9	67
Fi30	*Clostridiales sp. DTU058*	2.9	99	Ba02	*Rikenellaceae sp. DTU002*	1.9	88
Pr01	*Gammaproteobacteria sp. DTU037*	2.8	96	Ba01	*Rikenellaceae sp. DTU001*	1.9	95
Eu04	*Methanosarcina sp. DTU009*	2.8	95	Fi17	*Clostridia sp. DTU026*	1.9	82
Ba06	*Porphyromonadaceae sp. DTU048*	2.7	84	Fi19	*Clostridiales sp. DTU053*	1.9	96
Fi65	*Pelotomaculum sp. DTU098*	2.6	97	Fi52	*Clostridiales sp. DTU083*	1.9	93
Fi67	*Clostridiales sp. DTU100*	2.6	80	Fi35	*Clostridiales sp. DTU064*	1.9	86
Fi09	*Syntrophomonas sp. DTU018*	2.6	97	Sy04	*Synergistales sp. DTU085*	1.9	93
Fi43	*Clostridiales sp. DTU074*	2.6	92	Fi53	*Clostridia sp. DTU084*	1.9	79
Fi28	*Clostridiales sp. DTU055*	2.6	91	Fi22	*Clostridia sp. DTU030*	1.8	94
Fi39	*Clostridiales sp. DTU069*	2.6	92	Eu03	*Euryarchaeota sp. DTU008*	1.8	98
Fi62	*Clostridia sp. DTU095*	2.5	88	Fi69	*Clostridiales sp. DTU071*	1.8	52
Fi08	*Syntrophomonas sp. DTU017*	2.5	88	Fi06	*Clostridia sp. DTU015*	1.7	90
Fi15	*Clostridiales sp. DTU024*	2.5	94	Ba05	*Porphyromonadaceae sp. DTU047*	1.7	88
Fi12	*Clostridia sp. DTU021*	2.5	87	Pr07	*Campylobacterales sp. DTU103*	1.7	86
Fi51	*Clostridiales sp. DTU082*	2.5	75	Fi33	*Clostridia sp. DTU062*	1.7	79
Fi57	*Clostridiales sp. DTU089*	2.5	92	Fi29	*Bacilli sp. DTU057*	1.7	98
Pr10	*Alcaligenaceae sp. DTU106*	2.4	87	Sp02	*Treponemaceae sp. DTU108*	1.7	71
Fi34	*Tepidanaerobacter sp. DTU063*	2.3	95	Fi02	*Clostridia sp. DTU011*	1.7	83
Ba03	*Porphyromonadaceae sp. DTU003*	2.3	84	Fi11	*Clostridiales sp. DTU020*	1.7	71
Pr11	*Desulfobulbaceae sp. DTU107*	2.3	86	Fi42	*Clostridiales sp. DTU073*	1.7	93
Pr06	*Alcaligenaceae sp. DTU102*	2.3	76	Fi23	*Clostridiales sp. DTU031*	1.6	82
Fi07	*Syntrophothermus sp. DTU052*	2.3	97	Fi24	*Clostridiales sp. DTU032*	1.6	89
Fi05	*Clostridia sp. DTU014*	2.3	94	Fi41	*Clostridiales sp. DTU072*	1.6	96
Fi68	*Clostridiales sp. DTU101*	2.2	75	Sy02	*Synergistaceae sp. DTU044*	1.6	85
Fi20	*Clostridiaceae sp. DTU054*	2.2	91	Sy03	*Synergistaceae sp. DTU045*	1.5	92
Fi66	*Clostridiales sp. DTU099*	2.2	88	Ba07	*Rikenellaceae sp. DTU049*	1.5	68
Fi36	*Clostridia sp. DTU065*	2.2	94	Fi46	*Clostridia sp. DTU077*	1.5	68
Pr04	*Gammaproteobacteria sp. DTU040*	2.2	91	Fi26	*Clostridiales sp. DTU035*	1.5	90
Fi38	*Clostridia sp. DTU068*	2.2	93	Te02	*Acholeplasmatales sp. DTU061*	1.5	87
Fi55	*Clostridiales sp. DTU087*	2.2	94	Fi25	*Clostridiales sp. DTU033*	1.5	93
Fi47	*Clostridiales sp. DTU078*	2.2	91	Te03	*Acholeplasmatales sp. DTU067*	1.5	94
Fi10	*Syntrophomonas sp. DTU019*	2.2	91	Th01	*Thermotogaceae sp. DTU111*	1.4	82
Fi31	*Clostridiaceae sp. DTU059*	2.2	94	Fi58	*Clostridiales sp. DTU090*	1.4	75
Eu01	*Methanoculleus sp. DTU006*	2.2	93	Fi50	*Clostridiales sp. DTU081*	1.4	71
Fi18	*Peptococcaceae sp. DTU027*	2.1	93	Fi27	*Clostridiales sp. DTU036*	1.4	77
Fi37	*Clostridiales sp. DTU066*	2.1	90	Fi14	*Clostridiale sp. DTU023*	1.4	82
Fi04	*Clostridiales sp. DTU013*	2.1	89	Sy06	*Synergistales sp. DTU110*	1.4	55
Fi03	*Clostridiales sp. DTU012*	2.1	94	Sy01	*Anaerobaculum sp. DTU043*	1.4	59
Fi45	*Clostridiales sp. DTU076*	2.1	96	Eu05	*Methanothermobacter sp. DTU051*	1.2	78
Fi54	*Clostridiales sp. DTU086*	2.1	90	Te01	*Acholeplasmatales sp. DTU056*	1.2	95
Fi60	*Clostridiales sp. DTU092*	2.1	90	Tm01	*TM7 DTU050*	1.2	65
Sp01	*Spirochaeta sp. DTU042*	2.1	90	Fi56	*Clostridia sp. DTU088*	1.2	48
Fi64	*Clostridia sp. DTU097*	2.1	72	Fi59	*Erysipelothrix sp. DTU091*	1.1	96
Fi01	*Clostridiales sp. DTU010*	2.1	92	Te04	*Acholeplasmatales sp. DTU094*	0.8	85
Eu02	*Methanoculleus sp. DTU007*	2.0	97	Sy05	*Synergistaceae sp. DTU109*	0.8	57
Fi44	*Clostridiales sp. DTU075*	2.0	92	Fi63	*Eubacteriaceae sp. DTU096*	0.7	34
Fi61	*Clostridiales sp. DTU093*	2.0	89	Pr09	*Desulfomicrobium sp. DTU105*	0.7	30
Pr08	*Rhodocyclaceae sp. DTU104*	2.0	74	Th02	*Thermotogales sp. DTU112*	0.6	15

*Proteobacteria* is the second most abundant group (10 GBs), including *Alcaligenaceae* (Pr05, Pr06, Pr10), a group of three GBs that can only be assigned to the *Gammaproteobacteria* group (Pr01, Pr02, Pr04), two GBs belonging to the *Deltaproteobacteria* (Pr09, Pr11) and one belonging to the *Campylobacteraceae* (Pr07). *Alcaligenaceae* are not frequently reported in analysis of the biogas reactors [41, 42] and the analysis of their genomic composition can provide a first glimpse into their possible role. GB Pr09 has been tentatively assigned to the *Desulfomicrobium* group and it is one of the most interesting members of *Proteobacteria* because it is competing with methanogens in anaerobic enrichment cultures degrading oleate and palmitate [43, 44]. Finally, Pr11 is relevant because members of the *Desulfobacterales* are involved in acetate oxidation by parallel reduction of sulfur, a key process in the biogas microbial community [45].

*Bacteroidetes* is the third most abundant group (6 GBs) which is composed of two subgroups: *Porphyromonadaceae* (Ba03, Ba05, Ba06) and *Rikenellaceae* (Ba01, Ba02, Ba07). Both subgroups are dominant microorganisms in biogas plants [46, 47].

*Synergistetes*, which was a recently introduced phylum having only *Synergistaceae* family, was represented by 6 GBs in our study.

The remaining GBs are included in the phyla *Actinobacteria* (Ac01), *Thermotogae* (Th01, Th02), and *Spirochaete* (Sp01, Sp02). The abundance of all these GBs was low in the samples examined. Species of the phylum *Thermotogae* were identified also in thermophilic biogas-production plants utilizing renewable primary products for biomethanation, even at low abundances [48–50]. Their role in utilization of complex carbohydrates has been recently suggested on the basis of the gene content of *Defluviitoga tunisiensis* [51]. The low frequency of *Spirochaetes* is in agreement with relevant works in anaerobic digesters and their abundance seems to be highly variable depending on the operational conditions of the reactor [52]. Also, *Actinobacteria* have been previously reported at low abundance in biogas reactors [53, 54] but in the cited research their functional role was difficult to be predicted due to the lack of genomic sequences and their highly variable physiological and metabolic properties.

In the tree of life obtained using PhyloPhlAn, Tm01 was one of the most difficult taxonomic assignments as this GB was deeply branched from the candidate phylum *TM7* composed only by the *Candidatus Saccharimonas aalborgensis* [16]. Despite its genome that is not completely closed (72 % completeness), it is one of the most complete *TM7* reported in database and its small genome size (~1.2 Mbp) confirms data reported in the literature indicating that it is one of the smallest in the AD microbial community.

### Functional characterization of the biogas microbial community

In the cited literature, the role of the majority of microbial groups involved in biogas production has been hypothesized considering the functional characteristics of distantly related species. Nevertheless, the lack of genome sequences prevents a clear understanding of their physiology and behavior. Therefore, our analyses targeted to give answers to two questions; (a) how much specialized are the microorganisms, and (b) which are their roles in the metabolic pathways of AD process.

**Fig. 2:**
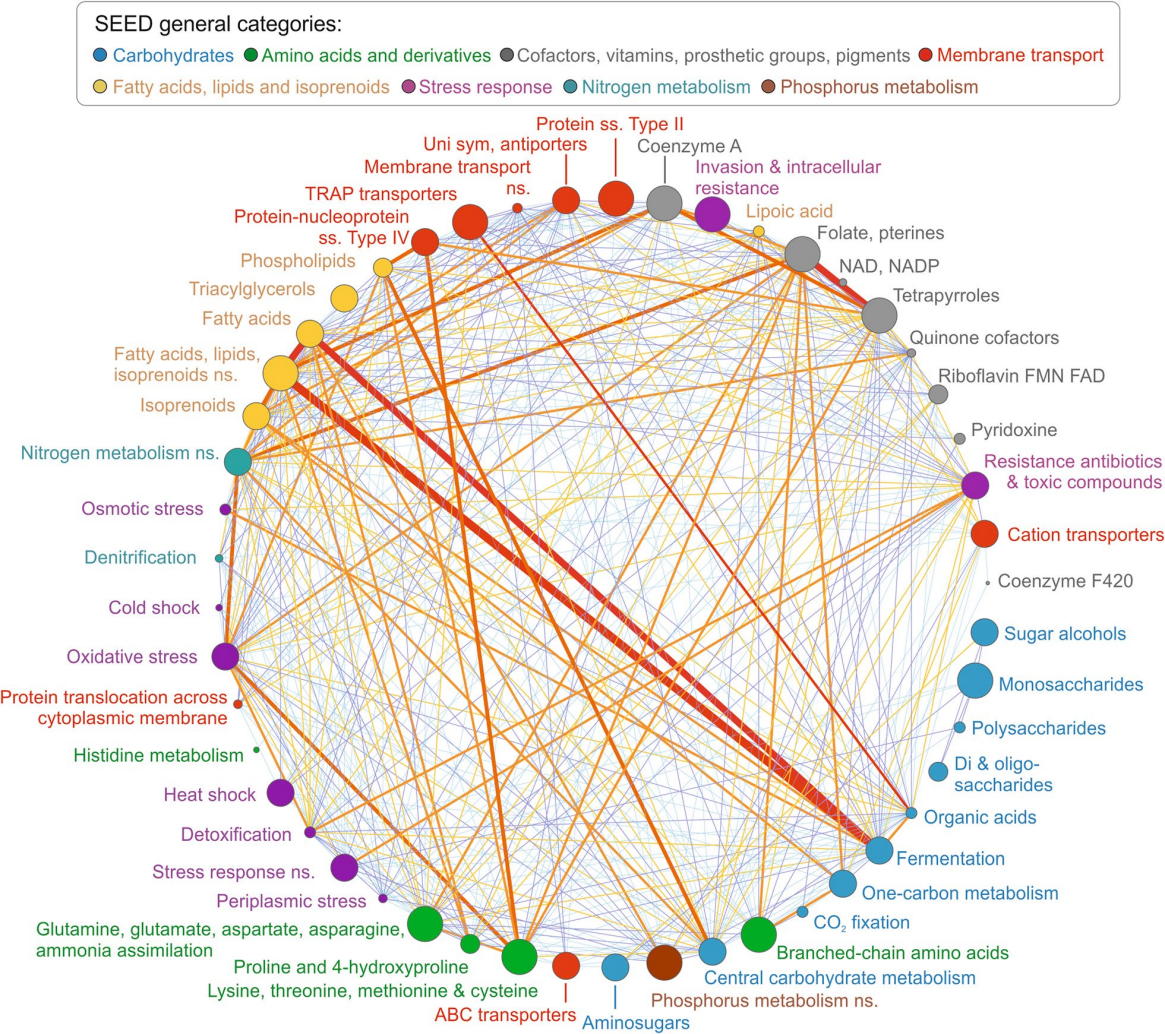
Network Representation of the Biogas Functional Organization (NRBFO). Nodes represent SEED functional categories. The size of each node is correlated to the number of GBs ranked among the top one-eighth of each functional category. *Edges thickness* is proportional to the number of GBs shared by two nodes; *edge colors* were used to simplify the visual observation of the connections. *Thick edges* connect nodes including GBs with high number of SEED feature counts in the two categories. Categories having *thin edges* are those comprising GBs that tend to have specialized functions

In order to elucidate the microorganisms’ specialization, we converted the results from SEED analysis (“Methods” and Additional file 1) into a Network Representation of the Biogas Functional Organization (NRBFO). For the construction of the NRBFO, we selected only the GBs that were ranked among the top 1/8 of each SEED functional category based on the number of corresponding genes. If one GB belonged in two categories, these were connected with an edge (Fig. 2; Additional file 4). This revealed that the GBs in the AD microbiome could be classified into two distinct groups according to their functional properties.

The first group consists of GBs specialized on a single metabolic process, as the ones enriched in genes belonging to the general functional category of “carbohydrate utilization and metabolism” (blue nodes in Fig. 2). More specifically, some GBs were only involved in “central carbohydrate metabolism,” others in “aminosugars utilization,” or “di- and oligosaccharides utilization,” and so on, generating a very complex and faceted organization inside the microbial community. On the contrary, the second group includes GBs possessing multifunctional roles (i.e., they have high number of genes in more SEED categories). These GBs are inside the nodes connected by thick edges in the network (Fig. 2). It was found that 10 GBs have high number of SEED feature counts both in “sugar fermentation” and “fatty acids oxidation,” Three of these GBs belong to *Syntrophomonadaceae* family (Fi07, Fi08, Fi09), two belong to *Alcaligenaceae* family (Pr05, Pr10), two to *Gammaproteobacteria* (Pr01, Pr02) and three to *Clostridia* class (Fi12, Fi62, Fi68) (Additional file 5). It is known that common functionalities can be shared by species of the same taxonomic group [11]. However, our analysis proved that in some cases, species of completely different taxonomic groups can share the same functional role and therefore compete for the same niche.

**Fig. 3:**
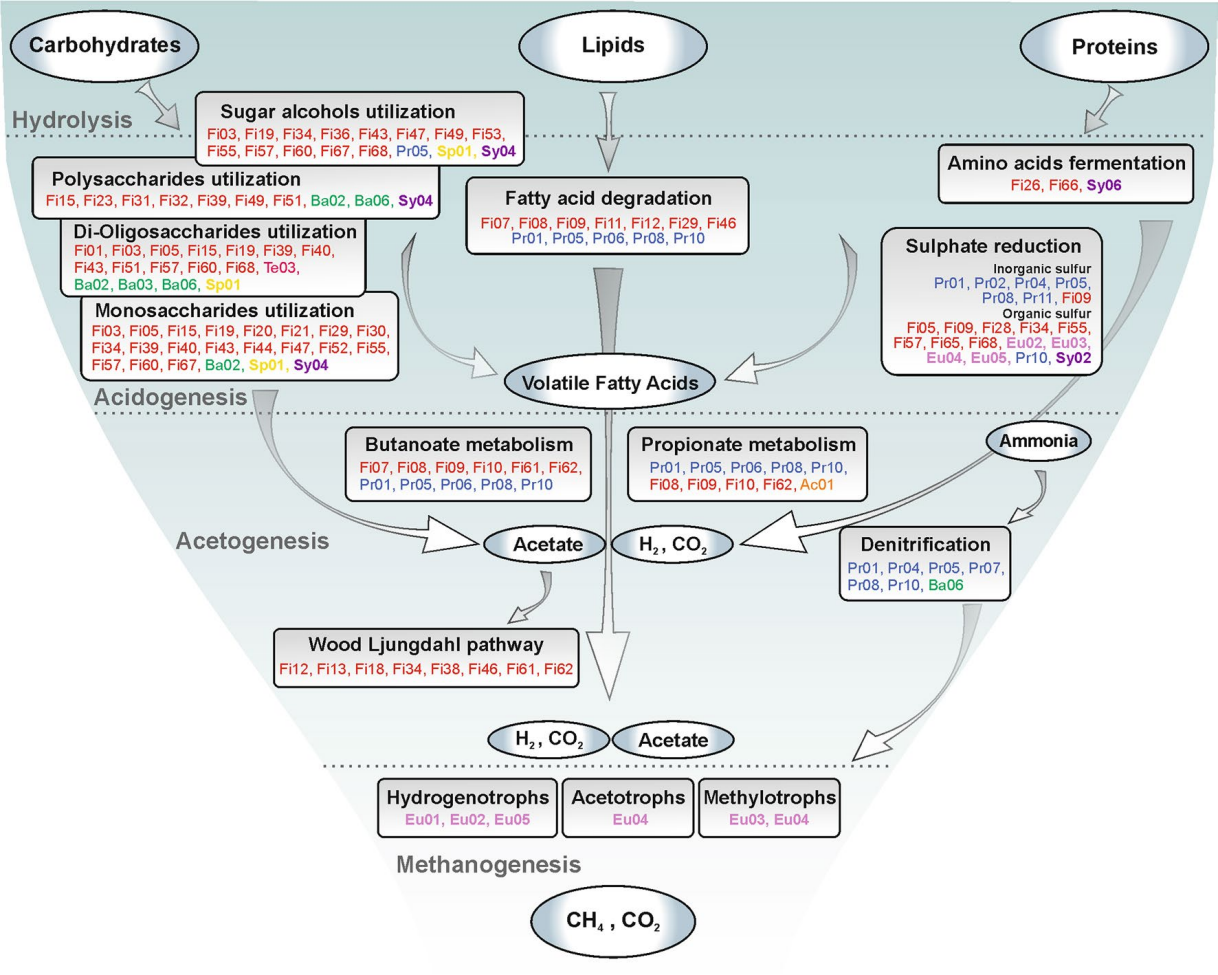
Functional roles of the GBs in the biogas production “food chain.” The main steps of the anaerobic degradation process are highlighted, together with the more relevant GBs involved. Functional roles were defined considering nearly complete KEGG pathways (Wood–Ljungdahl pathway, methanogenesis, propionate and butyrate metabolism), SEED categories (fatty acid degradation, carbohydrates utilization, denitrification, sulfate reduction), COG (amino acids fermentation) and Pfam (polysaccharides). Ovals refer to the compounds used by the microbial community (carbohydrates, fatty acids, proteins), intermediates (volatile fatty acids (VFA)-propionate, butyrate), and final products (carbon dioxide and methane)

As an additional step, the species were functionally classified considering the proposed organization of the AD process, which is divided in four layers (i.e., hydrolysis, acidogenesis, acetogenesis, and methanogenesis) (Fig. 3; Additional file 1: Figures S4–S7; Additional files 5, 6, 7, 8). In order to do this, a putative functional role for the GBs was assigned taking into account their annotation obtained by COG, KEGG, SEED, and Pfam (Additional file 1). The assignment showed that the AD microbiome bears resemblance to a funnel concept; during the initial step of organic substrate degradation (i.e., carbohydrates, proteins, and lipids), a wide variety of GBs (even belonging to different phyla) are involved. In contrast, while proceeding to the next steps of the AD process (i.e., acetogenesis, acidogenesis, and methanogenesis), the involved GBs become gradually more specialized.

Particular attention was drawn to key functional steps of the AD process in order to elucidate the role of GBs. For example, proteins involved in polysaccharide degradation are important as it is well known that the raw manure contains a high fraction of fibers due to animal nutrition. These proteins were identified using the SEED annotation (polysaccharides category) and also by selecting those with significant matches to at least one of the carbohydrate-binding modules proposed by Hess et al. [55]. Analysis of the Pfam domains was performed in order to minimize the dependence on the overall sequence similarity of candidate genes to known carbohydrate-active enzymes. Out of 7161 carbohydrate-binding proteins found in the global assembly, 1896 of them (~26 %) were assigned to specific GBs. Most of the GBs with high number of carbohydrate-binding modules belong to *Clostridiales* and considering the similarity of the 107 essential genes obtained using BLAST, can be related to *Ruminoclostridium* or *Clostridium*. These genera are well known for their involvement in polysaccharides degradation, and some species have been previously isolated in biogas plants [56]. The carbohydrates utilization process involves numerous species which are specialized in degradation of different carbohydrates groups (Fig. 3). These microorganisms cooperate with species involved in lipids or proteins degradation to generate the byproducts for the subsequent steps of methanogenesis.

In the fermentation of sugars to organic acids, an important role is played by the Wood-Ljungdahl pathway (WLP), which is characteristic for some acetogenic bacteria and archaea [57]. In this process, carbon dioxide is reduced to carbon monoxide and then converted to acetyl-CoA, with hydrogen serving as electron donor. From KEGG analysis performed on selected genes of the WLP, it was found that a specific subset of 8 bacterial species (Fig. 3; Additional file 1: Figure S6) features a complete or nearly-complete pathway. All these bacteria were assigned to *Firmicutes* and more specifically to *Clostridia* sp. (Fi12, Fi13, Fi38, Fi46, and Fi62), *Clostridiales* sp. (Fi61), *Peptococcaceae* sp. (Fi18), and *Tepidianaerobacter* sp. (Fi34). It is known that specific microbes are capable to perform also the reverse WLP (i.e., the so called Syntrophic Acetate Oxidation, SAO), which includes the same genes of the WLP. By this pathway, they oxidize acetate to hydrogen and carbon dioxide when growing syntrophically with hydrogenotrophic methanogens that utilize the hydrogen and carbon dioxide produced to generate methane [58]. The overall process can be viewed as an additional mechanism of methane formation from acetate, and was originally proposed by Barker [59] and later confirmed by Zinder and Koch [58]. The mechanism was initially described in thermophilic anaerobic processes [58, 60, 61] and was later on identified to occur also in reactors operating at mesophilic temperatures [62–64].

Another finding is related to the synergistic behavior between *Synergistetes* with other microorganisms. SEED subsystem revealed that the most similar sequenced species to Sy02, Sy03, Sy05, and Sy06 is *Thermanaerovibrio acidaminovorans* DSM 6589 and for Sy01 is *Anaerobaculum hydrogeniformans* ATCC BAA-1850. Therefore, the presence of numerous ABC transporters for branched-chain amino acids (AA) (Additional file 5) together with the large number of genes involved in AA metabolism (Additional file 1) indicates that *Synergistetes*, similarly to *T. acidaminivorans*, could operate synergistically with other species, to ferment AAs to acetate and propionate [65].

### Archaeal community characterization

**Fig. 4:**
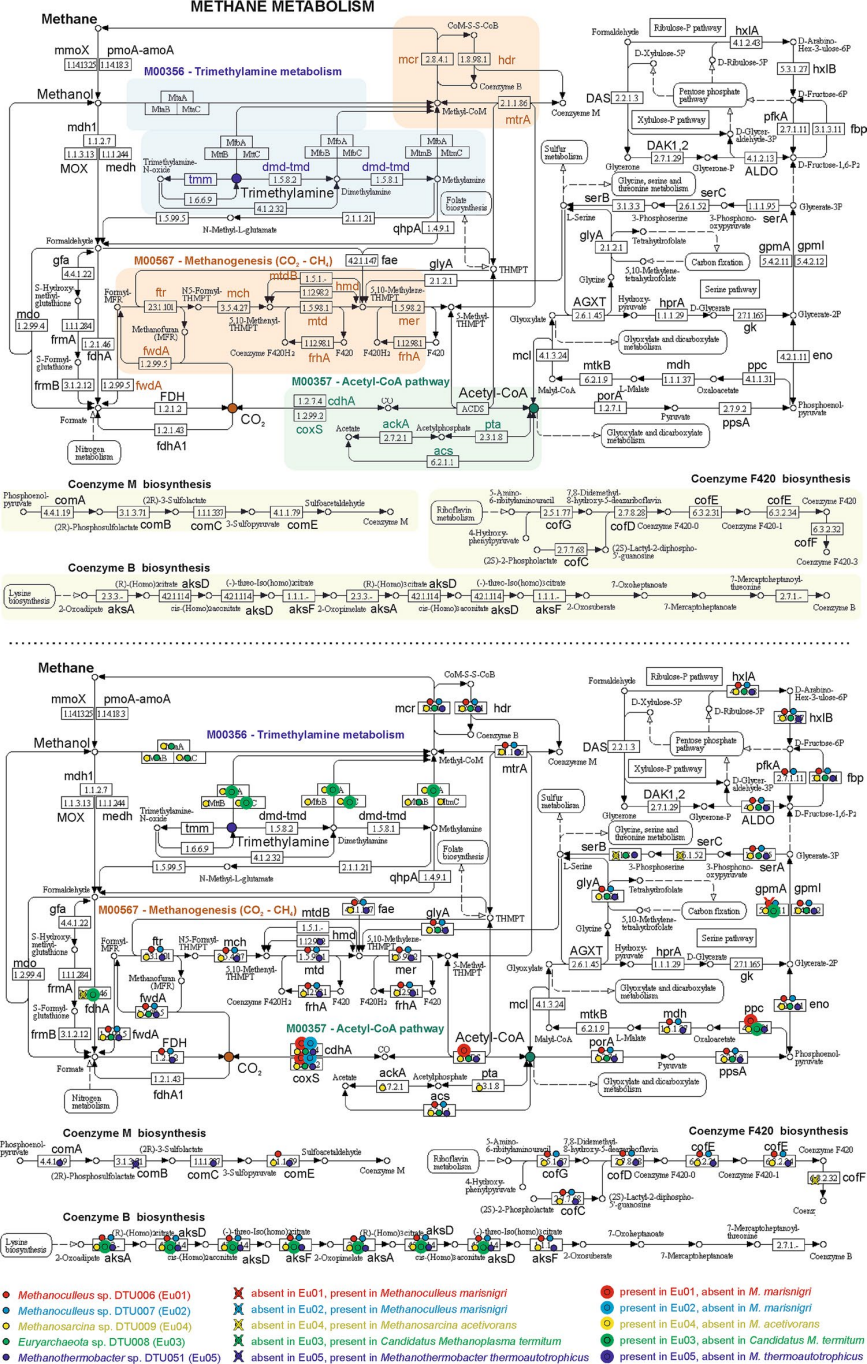
Comparison of the KEGG methane pathways of the 5 archaeal GBs (Eu01–05). In the *upper part* of the figure the reference KEGG methane metabolism pathway is represented, in the *lower part* archaeal GBs’ genes present and absent in the pathway are *highlighted*. Genes identified in the archaeal GBs were labeled with a *small colored dot*. Genes absent in the GBs and present in the reference genomes are marked with a “X” (Eu01–Eu02—*Methanoculleus marisnigri*; Eu03—*Candidatus Methanoplasma termitum*; Eu04—*Methanosarcina acetivorans*; Eu05—*Methanothermobacter thermoautotrophicus*). Genes identified in the GBs and absent in the reference are labeled with a *circled dot*

As previously discussed, the archaeal species are the best characterized in the biogas community. Archaea are dominated by the hydrogenotrophic methanogen Eu01 belonging to the *Methanoculleus* genus. Eu01, together with Eu02 (another *Methanoculleus* sp.), features all the central enzymes and complexes of methanogenesis: Mcr, Mtr, Fpo, and Hdr (Fig. 4). In addition, they feature all the complementary genes necessary for the reduction of CO_2_ to methane: *fmd*/*fwd*, *ftr*, *mch*, *mtd*, and *mer*. On the contrary, they both lack the gene phosphate acetyltransferase (*pta*), involved in the conversion of acetate to methane (aceticlastic pathway) and also all the genes coding for the methylamine and methanol corrinoid proteins, essential for the conversion of methyl groups from methanol and methylamines to methane (methylotrophic pathway).

**Fig. 5:**
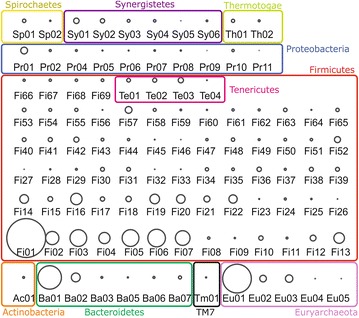
Graphic representation of the GBs abundance in the biogas microbial community. The GBs coverages are represented as *circles* where the area is proportional to the coverage. GBs are grouped considering the taxonomic assignment at phylum level (Sp *Spirochetes*, Sy *Synergistetes*, Th *Thermotogae*, Pr *Proteobacteria*, Fi *Firmicutes*, Te *Tenericutes*, Ac *Actinobacteria*, Ba *Bacteroidetes*, Tm *TM7* phylum, Eu *Euryarchaeota*). *Outlines colors* correspond to those reported in Fig. 1

Eu04, belonging to the *Methanosarcinales* genus, has a very low abundance (Fig. 5). It features all the genes belonging to the methylotrophic and aceticlastic pathway. Interestingly, it lacks the gene coding for Mtd, which catalyzes the fourth reaction in the reduction of CO_2_ to methane. As stated before, Eu01 and Eu02 are instead able to perform all the reactions of this pathway, and the fact that these two GBs are approximately 4000 and 270 folds more abundant than Eu04 (Fig. 5) is an indication that the hydrogenothrophic pathway is the most favorable at the tested conditions. This finding is in accordance with several studies performed in similar conditions [5, 6, 10].

Interestingly, the second most abundant archaeon is represented by a completely new *Euryarchaeota* (Eu03). It is remarkable that Eu03 was the second most abundant archaeal species in the microbial community. It has very small genome size (~1.76 Mbp) similarly to *Candidatus Methanoplasma termitum* and *Ca*. *Methanomassiliicoccus intestinalis* (1.48 and 1.93 Mbp, respectively). Comparative analysis of the methane pathway (performed on all the archaeal GBs) using KEGG (Fig. 4) revealed that, differently from the other archaeal GBs, Eu03 completely lacks the coenzyme F420 biosynthesis pathway. This feature is also evident in the recently sequenced *Ca. M. termitum* [66]. Interestingly, it was possible to identify pivotal methanogenic genes in Eu03, including some belonging to the methylotrophic pathway, which are instead absent in the *Methanomassiliicoccales* species previously sequenced. Due to the small number of archaeal genomes in public databases, all the taxonomic analyses performed on the essential genes (Additional file 3) failed to assign Eu03 to any previously defined lineage. Only by using BLASTP similarity search and analysis of the 16S rRNA, it was possible to identify a distant correlation with the recently discovered seventh order of methanogens, the *Methanomassiliicoccales* (previously referred to as “*Methanoplasmatales*”).

It is important to highlight that this new uncultured archaeon (Eu03) was identified, quantified, and assigned to a putative functional role only thanks to the binning strategy, which is the fundament of the present work.

## Conclusions

This study demonstrated that the metagenomic assembly and binning of the shotgun sequences obtained from biogas reactors allowed the identification of 106 GBs that can be assigned into the context of the biogas degradation food chain by means of bioinformatic analysis. This is a major step forward in the characterization of the biogas microbial community especially when compared to previous studies, where the functional roles have been inferred from those assigned to the more similar species identified considering 16S rRNA similarity. In the case of the biogas microbial community, the identified GBs are distantly related to species for which the genomes are available in the databases and, as previously discussed, a predictive metagenomics approach is not accurate. This is clearly demonstrated by the high fraction of new GBs identified and assigned only at high taxonomic level, as for example the newly identified methanogenic archaea (Eu03). Another concluding remark drawn by the binning process revealed that approximately 70 % of the assembly cannot be assigned to a specific GB. This suggests the presence of more than 450 GBs in the biogas microbial community. As this is the first attempt to deeply characterize the AD microbiome, it is expected that further studies performed under different operational conditions (e.g., different temperatures and substrate) will allow in the next future to enrich the genome database. Finally, this study opens new avenues in deciphering the functional interactions between microbial species involved in the AD process and provides a solid reference that will greatly simplify further metatranscriptomics and metaproteomics analyses.

## Methods

### Biogas reactors’ configuration

Eight laboratory scale Continuous Stirred Tank Reactors (CSTR) operating at thermophilic conditions (54 ± 1 °C) were selected for sampling as shown in Additional file 1: Figure S1. The influent substrate of the reactors was cattle manure with varying chemical composition. The reactor’s operating temperature and influent feedstock composition were chosen to resemble typical conditions occurring in centralized full-scale biogas plants. The Organic Loading Rate (OLR) of all reactors varied between 1.9–2.9 gVS/L reactor-day and the hydraulic retention time was kept constant at 15 days. The initial inoculum used derived from Snertinge biogas plant, Denmark.

### Sample collection

Eighteen samples for microbial analyses (~15 ml each) were collected at various times during the operation of the reactors (Additional file 9). The samples were denoted with the name of the reactor followed by a letter to designate the sampling time period (e.g., CSTR01a and CSTR01b).

### DNA extraction

Barley residues present in the manure were removed by filtering with a 100-μm nylon cell strainer filter. The filtered sample was centrifuged at 5000 rpm for 10 min and the supernatant was discarded leaving ~2 g of material. Genomic DNA was extracted from these 2 g of material using the RNA PowerSoil^®^ DNA Elution Accessory Kit (MO BIO laboratories, Carlsbad, CA, USA). The quality and the quantity of the extracted DNA were determined both using NanoDrop (ThermoFisher Scientific, Waltham, MA, USA) and Qubit fluorometer (Life Technologies, Carlsbad, CA, USA).

### Metagenome sequencing

Genomic DNA extracted from the samples was prepared for sequencing using two different procedures. A pool obtained using identical quantities of the samples CSTR01a, CSTR02a, CSTR03a, CSTR01b, CSTR02b, and CSTR03b was used to prepare libraries using TruSeq DNA PCR-free Kit v2 (Illumina, San Diego, CA, USA). Nextera DNA Library Preparation Kit (Illumina, San Diego, CA, USA) was used to prepare libraries for all the individual samples from the reactors (Additional file 1: Figure S1). All the samples (both pooled and individual) were paired-end sequenced (2 × 150 bp) using Illumina HiSeq 2500 (Illumina, San Diego, CA, USA). One lane of the sequencer was allocated to the pooled sample prepared with the TruSeq kit (~250 millions filtered reads) and one to the samples prepared using the Nextera kit (from 26–58 millions filtered reads for each sample). The TruSeq DNA PCR-free kit was used due to its superior coverage of DNA regions, which are traditionally difficult to sequence, such as high GC-rich regions. The sequences obtained from samples CSTR01a, CSTR02a, CSTR03a, CSTR01b, CSTR02b, and CSTR03b were assembled since they represented more than 50 % of the total obtained reads.

Sequence data reported in this study have been submitted to the National Center for Biotechnology Information (BioProject PRJNA283298). Raw sequence data have been deposited at Sequence Read Archive under accession SRP058179 and Whole Genome Shotgun projects have been deposited at DDBJ/EMBL/GenBank under the accession LFRM00000000-LFTS00000000. The versions described in this paper are the first version LFRM01000000-LFTS01000000.

### Reads trimming and de novo metagenome assembly

Reads in FASTQ format were quality-filtered and the adaptors were removed using Trimmomatic software [67]. Overlapped paired-ends were merged using Flash [68] with standard parameters, except from the maximum overlap parameter, which was set to 150. Assembly was performed using both paired-end reads (with insert size equal to 470 bp for TrueSeq and 280 bp for Nextera) and single-end reads (both those merged using Flash and those which only one end passed the filtering step). Reads were imported to CLC Genomics workbench v. 5.1 (CLC Bio, Aarhus, DK, USA) and assembled using CLC’s *de novo* assembly algorithm, using a kmer of 63, a bubble size of 60 and a minimum scaffold length of 500 bp.

### Gene finding and annotation

Gene finding on the scaffolds obtained from the assembly was performed using Prodigal, run in metagenomic mode [69]. Conserved protein families and domains were identified using reverse position-specific BLAST algorithm (RPSBLAST of NCBI BLAST+) performed on all predicted proteins, and using COG only [22] and Pfam [24] RPSBLAST databases. Only results with e-value lower than 1e-5 were considered, and additionally for COG only the best match was considered. KEGG annotation was performed using usearch7.0.1090_i86linux32 (-ublast) on the KEGG Orthology (KO) database [23] with e value cutoff 1e-5 (-maxhits 1) (http://www.drive5.com/usearch/). From the output file, KEGG pathway modules were identified using KOBAS [70]. After the binning process, scaffolds assigned to each GB were re-annotated via Rapid Annotation using Subsystem Technology (RAST) server [71]. The entire protein set of the five archaeal GBs were analyzed using KEGG Automatic Annotation Server (KAAS) [72].

### Taxonomic and functional analysis of the metagenome assembly

All the scaffolds obtained from the shotgun assembly were uploaded to the MG-RAST metagenomics analysis server [73] and analyzed using standard parameters, except from the minimum alignment length that was set to 100 bp. It should be noted that scaffold coverage was not assigned at the uploading. Results were visualized using KRONA software [74].

Taxonomic assignment of the GBs was performed with four different methods, and the detailed procedure is reported in the Additional file 1; results were then compared to extract the best possible one (Additional file 3). Briefly, the essential genes associated to each GB were checked by sequence similarity to the NR database using BLASTN, with e-value threshold 1e-5. The taxonomic assignment of the best match was recovered and sequence similarity of 95, 85, and 75 % or better was used for species, genus, and phylum level taxonomical assignment [17], respectively. A similar analysis was performed using BLASTP. The phylogenetic result and the microbial tree of life (Fig. 1; Additional file 10) were determined using Phylophlan [75] and the scaffolds of each GB were analyzed using Phylopythia [76].

Taxonomy was determined from NR database alignment, while functional classification was determined using COG [22] and SEED [71] (Additional file 1: Figure S8). Results are available at the MG-RAST database (meta-assembly) under accession number 4636806.3.

### Hypergeometric analysis

Hypergeometric analysis was performed to calculate the probability of observing the number of genes belonging to a specific functional category in each GB [77]. The probability *P* of finding at least *k* genes of a specific functional category within a group of *n* genes (the total number of genes of a GB) is given by

$$P = \sum\limits_{i = k}^{n} {\frac{{\left( \begin{aligned} f \hfill \\ i \hfill \\ \end{aligned} \right)\left( \begin{aligned} g - f \hfill \\ n - i \hfill \\ \end{aligned} \right)}}{{\left( \begin{aligned} g \hfill \\ n \hfill \\ \end{aligned} \right)}}}$$

where *f* is the total number of genes of a specific functional category determined considering all the GBs together, *g* is the total number of genes determined in all the GBs. Finally, we recursively repeated the calculation on SEED functional categories, KEGG pathways, and finally on COG functional categories. All the statistical calculations were performed using the R package.

### Calculation of the scaffold coverage

Reads obtained individually (using the Nextera kit) for 18 samples collected from all the reactors were aligned on the scaffolds larger than 500 bp with Bowtie2 software [78] and scaffold coverage was determined with the genomecov software of the BEDTools package [79]. Coverage was normalized considering the number of aligned reads and using the sample with the lower number as a reference. The coverage obtained was considered both for comparison between the number of genes of each KEGG pathway and its average coverage (Additional file 1 and Additional file 1: Figure S2), and also for the “refinement” of the binning process (“Methods” “Binning refinement through identification of co-abundant scaffolds,” a more detailed description is reported in Additional file 1).

### Identification of conserved marker genes

A set of 107 Hidden Markov Models of essential single-copy genes [32] were searched against the predicted open reading frames using HMMER3 (http://hmmer.janelia.org/) [80], following the strategy of Albertsen et al. [16]. The results were used to predict completeness and level of duplication of the GBs identified using the script “determine_bins_completenes.pl.”

### Identification of 16S rRNA genes

Scaffolds encoding the 16S rRNA genes were identified using the method described by Albertsen et al. [16]. Taxonomical assignment of the 16S rRNA genes was determined using RDP classifier [81] with a confidence threshold of 0.8.

### Binning of genomes using tetranucleotide composition and coverage

Initial binning was performed using the procedure of Albersten et al. [16] which is based on “sequence composition-independent binning and tetranucleotide binning.” During the first step, distinct groups of scaffolds were identified based on their coverage similarity in a pair of samples. During the second step, principal component analysis of tetranucleotide frequencies was used to separate species present in the same coverage-defined GBs.

### Binning refinement through identification of co-abundant scaffolds

The GBs extracted with the procedure described above are of high quality and were used as “internal controls” to verify the binning procedure based on the coverage strategy. MeV software [82] was used to examine the coverage profile of the scaffolds, which contain the essential single copy genes and are assigned to the GBs in all the 18 samples. Using Euclidean distance calculation (single linkage) on the coverage profiles of the scaffolds, the GBs were separated and manually checked. Visual analysis of the clusters previously assigned to the GBs allowed the selection of those that were clearly separated from the others. Subsequently, each group of scaffolds was used to generate a “canopy profile.” Each profile was used to extract (from the entire list of scaffolds) those having Euclidean distance smaller than 1 SD from the distribution of the “canopy scaffolds.” This step was performed using the script “extract_scaffold_euclidean.pl.” Finally, the paired-end connections between scaffolds were used to assign scaffolds to the GBs using the procedure reported by Albersten et al. [16]. Due to the high number of GBs, the procedure was not performed “manually” but implemented in the script “recover_interacting_scaffold.pl.” To minimize the misassignments, only scaffolds having average coverage within threefolds from the interacting scaffold and having a number of paired-end connections of at least 1/3^rd^ of the scaffold average coverage were considered. Genome contamination, which can inflate genome completeness estimates, was determined both by checking the number of essential genes present in more than one copy on a single GB and also using CheckM software [33]. More details regarding the binning procedure are reported in Additional file 1 (Binning strategy). The extracted GBs and the scripts used for binning refinement can be downloaded from http://www.biogasmicrobiome.com together with the manual and the test files. MetaBAT [21] was used in order to evaluate the performance of the binning strategy proposed in the current manuscript. The software was executed using default parameters but also “–sensitive” and “–specific.” The results reported refer to the “–sensitive” test.

### Recovery of the multifasta files and of the protein sequences encoded by the GBs

Using the IDs of the scaffolds, it was possible to recover the multifasta file from the entire metagenome assembly multifasta using the script “extract_sequences_from_fasta.pl.” Moreover, with the same script, it was possible to extract the protein sequences from the fasta file containing all the proteins predicted using Prodigal software [69].

## Additional files

10.1186/s13068-016-0441-1 Additional text file that contains supplementary information regarding: assembly, gene finding and annotation, comparison between number of genes belonging to each KEGG pathway and coverage, binning strategy, taxonomic assignment of the GBs, functional roles of the microbial species, number of genes for each KEGG pathways modules identified in the genome bins, number of genes identified in the genome bins for some selected KEGG pathways, methanogenic archaea.
10.1186/s13068-016-0441-1 Gene annotation. Annotation of the genes identified in the assembly using COG, KEGG and Pfam databases.
10.1186/s13068-016-0441-1 Taxonomy assignment and characteristics of the GBs. The taxonomy of the GBs identified was determined using different methods and a taxonomic assignment was suggested considering the results obtained. In columns (A–V) are reported: (A) the acronym of the GBs as reported in the main text (Sp = Spirochetes; Sy = Synergistetes; Th = Thermotogae; Pr = Proteobacteria; Fi = Firmicutes; Te = Tenericutes; Ac = Actinobacteria; Ba = Bacteroidetes; Tm = TM7 phylum; Eu = Euryarchaeota) the Phylum was determined using Phylophlan and, secondly, the results obtained from BLASTP search versus nr databases filtered using MEGAN; (B) the tentative taxonomic assignment; (C) domain of the genome bin; (D) phylum; (E) taxonomic level considered for the name assignment (the result obtained using Phylopythia was used when more than 50 % of the genome bin sequence was assigned to the same taxonomic group); (F) confidence for taxonomic assignment obtained using Phylophlan; (G–I) domain, phylum, class determined using Phylophlan; (J) taxonomic assignment determined using Phylopythia; (K) percentage of the genome assigned as reported in “J”; (L) taxonomic level reported in “J”; (M) number of genes having BLASTP e-value lower than 1*E-5; (N) average similarity for BLASTP results; (O) number of genes having BLASTN e-value lower than 1*E-5; (P) average similarity for BLASTN results; (Q) the species having the highest number of best match in BLASTP column “M”; (R) taxonomy assignment obtained using RDP classifier on the 16S rRNA gene, similarity, contig where the 16S gene was identified; (S) total length of the scaffolds assigned to the genome bin; (T) number of scaffolds, (U) scaffolds N50, (V) scaffolds N90, (W) average scaffolds length, (X) number of contigs determined after splitting scaffolds on stretched of 10 or more unknown bases “N”, (Y) contigs N50, (Z) contigs N90, (AA) average contigs length, (AB) number of protein encoding genes identified using SEED subsystem; (AC) number of protein encoding genes identified using Prodigal; (AD) total number of essential genes identified, (AE) univocal number of essential genes (removed those in multiple copies); (AF) estimated completeness of the GB; (AG) average number of essential genes in phylum 1. Bold text in columns (A, U, Y, Z, AA, AF) refers to GBs that satisfy the Human Microbiome Project quality criteria; (AH) estimated contamination level determined considering the univocal number of essential genes and the total number of essential genes in multiple copies; (AI) estimated completeness of the GB determined using CheckM software; (AJ) estimated level of contamination determined using CheckM software.
10.1186/s13068-016-0441-1 Input files used for the network Representation of the Biogas Functional Organization (NRBFO). Functional analysis of the microbial community begins with the identification of the GBs enriched in SEED subsystem counts (ranked among the top one eighth of each SEED category). Starting from the entire list of SEED sub-categories (Data Set 6) 56 were selected (the more relevant for the characterization of the biogas community) and the GBs with high number of subsystem counts were identified (“node abundance” worksheet). The subsystem categories were compared in order to identify if they shared the same GBs and the couples with three or more common genome bins were identified (“edge_size_connections” worksheet). The number of GBs identified for each category was used to set the node size and the number of GBs shared was used to set the edge size (Fig. 2).
10.1186/s13068-016-0441-1 Functional characterization of the GBs according to SEED. (“SEED” worksheet) GBs were annotated using SEED subsystem and for each category (row 1) the subcategories are reported (row 2). Numbers refer to the subsystem feature counts. The SEED categories are reported in columns highlighted in grey (C, R, X, AF, AH, AL, AP, AX, BN, BR, BV, BZ, CF, CJ, CO, CV, DG, DN, DT, DW, DZ, EH, ER, EW, FH, FL, FN). In columns at right of each category are reported the results for the subcategories (for example, columns D-Q refer to the “super-category” “C”). Rows 110–114 report the median, the third and the first quartile results for each column. In red and in blue are highlighted the GBs having high and low numbers of subsystem feature counts (those in the third and first quartile of each category). (“hypergeometric” worksheet) For each GB and each SEED functional category the P value obtained from hypergeometric distribution is reported.
10.1186/s13068-016-0441-1 Functional characterization of the GBs according to COG. (“COG_gene_numb” worksheet) GBs were annotated using COG. Numbers refer to the genes identified on each GB for each COG category. The COG categories are reported in columns (C–AA), the GBs are reported in rows (2–107). (“COG_perc” worksheet) Percentages of genes belonging to COG categories are calculated with respect to the total number of COG results for each GB (note that some genes belong to more than one COG category). In red and green are highlighted, for each COG category, the GBs having the 10 highest and the 10 lowest percentages. (“hypergeometric” worksheet). For each GB and each COG functional category, the P value obtained from hypergeometric distribution is reported.
10.1186/s13068-016-0441-1 Functional characterization of the GBs according to KEGG. (“KEGG” worksheet) GBs were annotated using KEGG. Numbers refer to the genes identified on each GB for each KEGG pathway. The KEGG pathways are reported in columns (C–EZ), the GBs are reported in rows (2–107). (“hypergeometric” worksheet) For each GB and each KEGG pathway, the P value obtained from hypergeometric distribution is reported.
10.1186/s13068-016-0441-1 Database resources used for functional characterization of the GBs. Functional processes of the GBs analyzed in the AD community are reported in the first column. The SEED subsystems, KEGG pathway maps, COG categories and Pfam domains used are reported in columns 3 and 4.
10.1186/s13068-016-0441-1 Metadata regarding the operational conditions of the reactors. Metadata regarding the operational parameters of the reactors including pH, methane yield and volatile fatty acid concentration.
10.1186/s13068-016-0441-1 Suggested viewer: FigTree http://tree.bio.ed.ac.uk/software/figtree/. Newick format of the file representing the microbial tree of life. The tree reports the 106 GBs of the AD microbial community together with other 3,737 microbial genomes. The file was obtained with PhyloPhlAn using 400 broadly conserved proteins used to extract phylogenetic signal.

## Abbreviations

AD: anaerobic digestion
AA: amino acid
COG: Clusters of Orthologous Groups
CSTR: continuous stirred tank rreactor
GB: genome bin
KAAS: KEGG Automatic Annotation Server
KEGG: Kyoto Encyclopedia of Genes and Genomes
KO: KEGG orthology
NRBFO: Network Representation of the Biogas Functional Organization
OLR: Organic Loading Rate
RAST: rapid annotation using subsystems technology
SAO: syntrophic acetate oxidation
WLP: Wood–Ljungdahl Pathway
