# Supplementary material for: Metagenomic analysis and functional characterization of the biogas microbiome using high throughput shotgun sequencing and a novel binning strategy
Source: Biotechnol Biofuels. 2016 Feb 2;9:26. doi: 10.1186/s13068-016-0441-1 (PMC4736482; doi:10.1186/s13068-016-0441-1)
Supplement: Supplementary file 1 — 10.1186/s13068-016-0441-1 Additional text file that contains supplementary information regarding: assembly, gene finding and annotation, comparison between number of genes belonging to each KEGG pathway and coverage, binning strategy, taxonomic assignment of the GBs, functional roles of the microbial species, number of genes for each KEGG pathways modules identified in the genome bins, number of genes identified in the genome bins for some selected KEGG pathways, methanogenic archaea. [file 13068_2016_441_MOESM1_ESM.pdf]

# Additional File 1

# Metagenomic analysis and functional characterization of the biogas microbiome using high throughput shotgun sequencing and a novel binning strategy

Campanaro S, Treu L, Kougias PG, De Francisci D, Valle G, Angelidaki I

Correspondence: P.G. Kougias, Department of Environmental Engineering, Technical University of Denmark, Kgs. Lyngby DK-2800, Denmark. E-mail: panak@env.dtu.dk. Tel: +45 45 25 14 54.

**Other Additional Materials for this manuscript includes the following:**

Additional Files 2 to 9: [Table S1. Annotation of the genes identified in the metagenomic assembly; Table S2. Taxonomy assignment and characteristics of the GBs; Table S3. Functional characterization of the GBs according to COG; Table S4. Functional characterization of the GBs according to SEED; Table S5. Functional characterization of the GBs according to KEGG; Table S6. Input files used for the network Representation of the Biogas Functional Organization (NRBFO); Table S7. Database resources used for functional characterization of the GBs; Table S8. Metadata regarding the operational conditions of the reactors]
Additional File 10. Newick format of the file representing the microbial tree of life reporting the 106 GBs of the AD microbial community together with other 3,737 microbial genomes. The file was obtained with PhyloPhlAn using 400 broadly conserved proteins used to extract phylogenetic signal.

## Contents

**Assembly, gene finding and annotation**

**Comparison between number of genes belonging to each KEGG pathway and coverage**

**Binning strategy**

**Taxonomic assignment of the GBs**

**Functional roles of the microbial species**

- **COG Analysis**
- **SEED analysis**

**Number of genes for each KEGG pathways modules identified in the genome bins**

**Number of genes identified in the genome bins for some selected KEGG pathways**

**Methanogenic archaea**

***Assembly, gene finding and annotation.***
Genomic DNA extracted from the microbial community was deeply sequenced to obtain a high quality representation of the biogas microbial community. The heterogeneous chemical composition of the reactors' influent feedstock (Figure S1) determined variations in abundance of the microbial species, allowing the assignment of the scaffolds to the GBs ("Binning strategy" section).
Considering that the global assembly contains 937,207 protein-encoding genes, and that 220,987 were assigned to the 106 GBs, it is possible to estimate that in the assembly a total of ~450 species could be represented. This is probably an under-estimation of the real number because only part of the genes is present in the assembly. Approximately 30% of the total reads could not be assembled, but it is difficult to translate this number into microbial species that could not be assembled, due to their extremely low abundance.

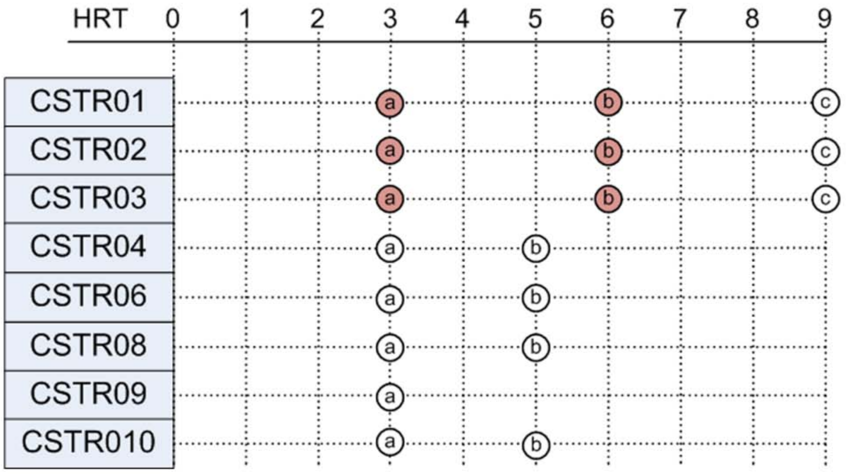


**Figure S1** Schematic representation of all the reactors used for samples collection. All the reactors had an HRT of 15 days and were operating at thermophilic conditions. (a), (b) and (c) represents sampling points. The samples marked in red were used for metagenomics assembly, all the samples were used to determine scaffold abundance used in the "clustering strategy". The samples and reactor names are as deposited in the sequence read archive under BioProject ID PRJNA283298.

**Table S1.** Number of reads obtained from the TruSeq and Nextera libraries for each sample after quality filtering. All the “TruSeq reads” and the “Nextera” paired reads combined with Flash software of the samples CSTR01a, CSTR01b CSTR02a, CSTR02b, CSTR03a and CSTR03b were used to generate the assembly. All the other “Nextera reads” were used for the binning process.

| **Sample** | **TruSeq** | **Nextera** | |
|---|---|---|---|
| | **Filtered Reads** | **Filtered Reads** | **% Reads Aligned** |
| *CSTR01a** | 254466426 | 48267271 | 67.67% |
| *CSTR01b** | | 58538728 | 70.12% |
| *CSTR02a** | | 53928985 | 68.27% |
| *CSTR02b** | | 57677194 | 70.40% |
| *CSTR03a** | | 56397104 | 57.03% |
| *CSTR03b** | | 56150684 | 70.04% |
| *CSTR01c* | na | 37228579 | 90.91% |
| *CSTR02c* | na | 37572549 | 91.51% |
| *CSTR03c* | na | 40310889 | 91.39% |
| *CSTR04a* | na | 47344505 | 64.81% |
| *CSTR04b* | na | 39312798 | 66.36% |
| *CSTR06a* | na | 44493386 | 66.92% |
| *CSTR06b* | na | 39594102 | 66.61% |
| *CSTR08a* | na | 39194561 | 67.64% |
| *CSTR08b* | na | 43827775 | 69.15% |
| *CSTR09a* | na | 37263499 | 64.12% |
| *CSTR10a* | na | 36430494 | 67.20% |
| *CSTR10b* | na | 26672721 | 73.38% |

*These samples were used to generate the assembly

***Comparison between number of genes belonging to each KEGG pathways module and coverage.***

Genes belonging to each KEGG pathways module are reported in the Additional File 2: Table S1 and details regarding the genes annotation procedure are reported in the Materials and methods section. For each KEGG pathways the total number of genes was determined. The average coverage for each class was determined for CSTR01a, CSTR02a and CSTR03a samples (chosen because they had similar manure composition and because they were operated for the same time before sampling) and was obtained aligning their sequencing reads on the global assembly using Bowtie2 software [1]; the coverage was calculated using the genomecov software of the bedtools package [2]. The average coverage was determined taking into account the total number of genes for each KEGG pathways module. In Figure S2 only the more relevant KEGG pathways modules are reported and they were ranked considering the ratio “coverage/number of genes”. Classes with the higher ratio are those more represented in the most abundant GBs.

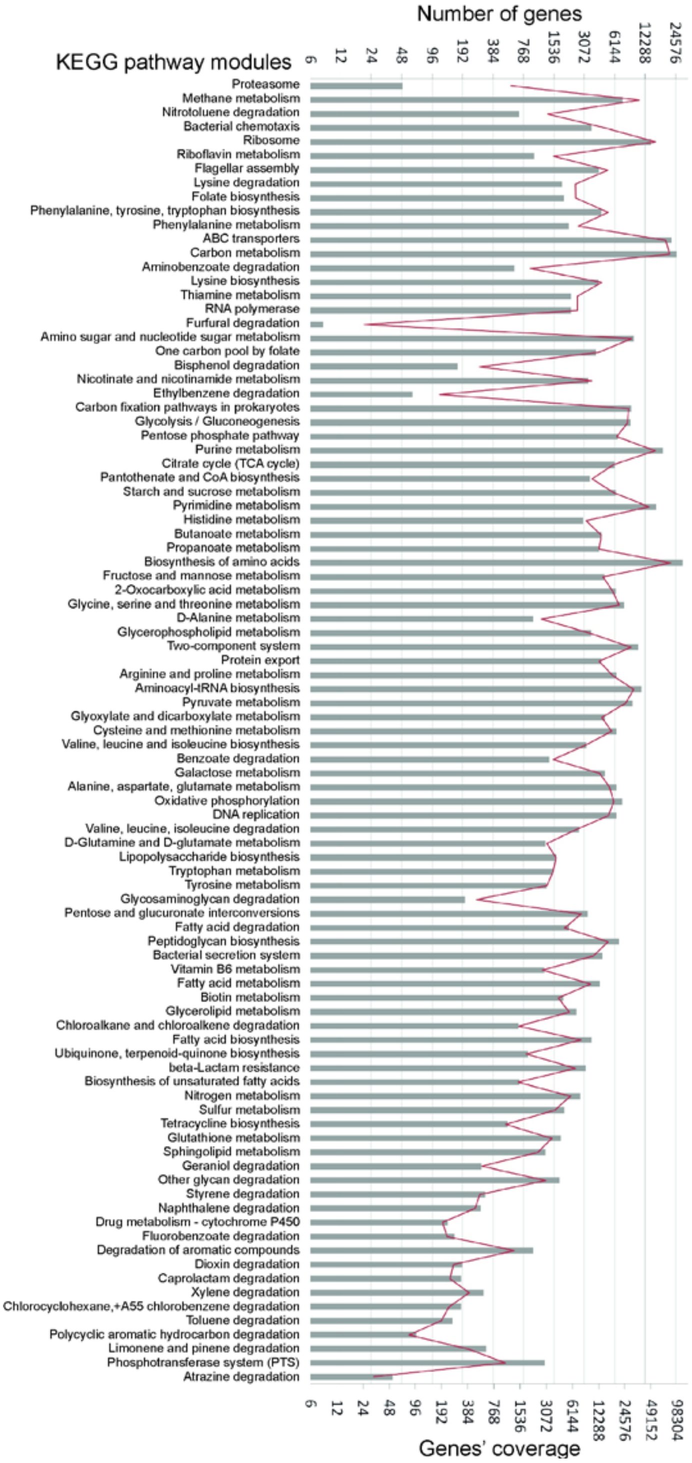


**Figure S2** Graph reporting the comparison between number of genes for each KEGG pathway module and their coverage. Histogram reports the number of genes (grey bars) and coverage is represented by the red line. Coverage is the average among samples obtained from bioreactors CSTR01a-03a. Only some selected KEGG pathways modules are reported.

***Binning strategy.***

Identification of the GBs was performed with a "two-steps strategy" (Figure S3). All the extracted GBs and the scripts used for the binning procedure can be downloaded from http://www.biogasmicrobiome.com.

In the first step, GBs were separated considering both their tetranucleotide composition and also a pairwise comparison of the scaffolds coverage; this procedure was performed by means of the "metagenome.workflow" script [3] (metagenome-workflow strategy). In this step, the scaffolds coverage determined in the first three CSTRs (samples 01a, 02a, 03a, 01b, 02b, 03b) were those considered for extraction of the GBs. In this procedure, different pairwise comparisons were considered for the binning.

Using the procedure described by Albertsen et al. [3], the essential genes were identified and taxonomically assigned considering the BLASTP results filtered using MEGAN software [4].

The second step of the clustering strategy was based on binning co-abundant scaffolds across all the metagenomic samples collected (Figure S1) (named "coverage strategy"). This procedure was implemented due to difficulties in the application of the "metagenome-workflow strategy" to numerous different samples. The main difficulty was due to the binning of the same GBs in different pairwise comparisons but also to the excessive time requested by the procedure. Despite these limitations, the GBs extracted from the metagenome workflow strategy are of high quality and useful to verify the rest of the binning procedure based on the coverage strategy. All the scaffolds encoding essential genes were extracted and their coverage in the 18 samples analyzed was recovered. To verify if the scaffolds assigned to each genome bin using the same procedure have similar behavior, the file was uploaded in MeV software [5] and clustered (Euclidean distance and single linkage). Clustering procedure cannot be applied to the entire population of 409,831 scaffolds because this would require an enormous computational effort. For this reason, the clustering procedure was applied only to the 12,345 scaffolds taxonomically assigned. Using MeV, a color was assigned to each GB (extracted in the first step) and to each of the 12,345 taxonomically assigned scaffolds. After the hierarchical clustering procedure, these colors allowed a simple verification of the correctness of the GBs extracted in the first step and of the presence of GBs not previously extracted with the metagenome workflow strategy. With this "coverage strategy" 61 additional GBs were identified. The script "extract_scaffold_euclidean.pl" was used to extract from the entire list those belonging to the GBs identified. This script calculates the average coverage profile (the "canopy profile") and the SD of the Euclidean distance for each GB identified with MeV software and then it extracts from the entire scaffold list those having Euclidean distance smaller than 1 SD from the canopy profile.

After these two steps, the paired-ends (PEs) connecting other scaffolds to those identified were recovered using a procedure previously proposed [3] (PE strategy). In this final process, the binned scaffolds (named "baits") allowed the recovery of others scaffolds (named "preys") using "recover_interacting_scaffold.pl". The preys can be recovered considering the PE connections, only if they have a coverage similar to the baits (ratio <= 3 folds or >= 0.333 folds) and if the number of PEs was >= to at least 1/3$^{rd}$ of the coverage of the nodes (i.e. at least 10 "PE connections" between a "bait scaffold" having coverage 30 and a prey scaffold are needed to collect the "prey" scaffold). The last parameter was introduced to avoid the recovery of high number of scaffolds connected by very low number of PE connections to the scaffolds with very high coverage (spurious connections).

All the parameters were chosen checking the procedure on the same GBs identified with the metagenome-workflow strategy and testing the completeness and the number of duplicated essential genes identified with the new procedure. At each step of the procedure, genome size of the bins, number of the essential genes and number of duplicated essential genes was

estimated using “extract_data_from_contigs_list.pl” in order to verify differences between the new strategy and the metagenome-workflow strategy. The final parameters used were those generating the best results in terms of GBs completeness and number of duplicated essential genes.
At the end of the binning all the scaffold lists (one for each bin) were merged into one single table using “compare_lists.pl”, and each scaffold was labeled as “cyto” (if it was recovered by the paired-end strategy) or “binned” (if it was recovered with the metagenome-workflow strategy or with the coverage strategy). The scaffolds assigned to more than one GB were discarded or retained on the basis of three rules. (1) They were removed from all the GBs if they were binned considering co-abundant genes coverage in two or more GBs, because they cannot be assigned due to this unreliable coverage. (2) The scaffolds were also removed if they were recovered by the paired-end strategy in more than one GB. (3) They were maintained if the scaffolds were assigned to two different GBs with different strategies (paired-end and coverage profile) but they were assigned only according to the coverage profile strategy (which is considered more reliable).
The selection process was performed using “separate_table_binning.pl” script which also generates multiple lists with scaffolds IDs (one for each GB) that can be used to obtain multifasta files used in the next steps.
Some GBs having high (>1000x), medium (500-1000x) and low (<500) coverage were selected for reassembly using only reads belonging to the GB scaffolds. Only in a few cases the reassembly process resulted in a reduction of the scaffold numbers; for this reason GBs were not reassembled at the end of the binning pipeline.
The entire binning procedure resulted in the identification of 115 GB; 9 were discarded due to the low level of estimated completeness or to the high number of duplicated genes. The completeness of the GBs and the amount of duplication was defined comparing the univocal and the total number of essential genes present on each GB. It was found that only 31 out of 107 essential genes are present in the archaeal GBs identified, but of course this is not due to incompleteness but to the absence of some essential genes in archaea or to the high level of divergence. To better evaluate the completeness of the five archaeal GBs a further analysis was performed with CheckM software [6] and it was found that only Eu05 has a completeness around 78%, all the others are more than 90% complete (Additional File 3: Table S2). It was also considered that not all the bacterial phyla harbor all the 107 essential genes [3] and this allowed a better evaluation of the GBs completeness. The average completeness of 83% (Additional File 3: Table S2) is slightly underestimated due to difficulties in estimating this value for archaea on the basis of the the107 essential genes. By using CheckM software, the completeness of the archaeal GBs was better evaluated, and the average completeness of the GBs was estimated around 85%.
For each GB the assembly result was checked by using the Human Microbiome Project assembly criteria [7]. The threshold used for each HMP criteria validation are: (1) contig N90 > = 500 bp (all GBs meet this criteria); (2) 90% or more of the essential genes are found (60 GBs meet this criteria, but also 4 *Euryarchaeota* are nearly complete); (3) contig N50 > = 5 kb (84 GBs meet this criteria); (4) scaffold N50 > = 20 kb (46 GBs meet this criteria); (5) average contig length > = 5 kb (57 GBs meet this criteria). The 35 GBs of high quality that meet all the criteria were highlighted in bold in Additional File 3: Table S2; 20 GBs meet at least 4 criteria, 14 GBs meet at least 3 criteria, 16 GBs meet at least 2 criteria and 21 GBs meet only one criteria. The 53 GBs satisfying at least 4 assembly criteria and the 5 archaeal GBs were submitted to NCBI WGS database.

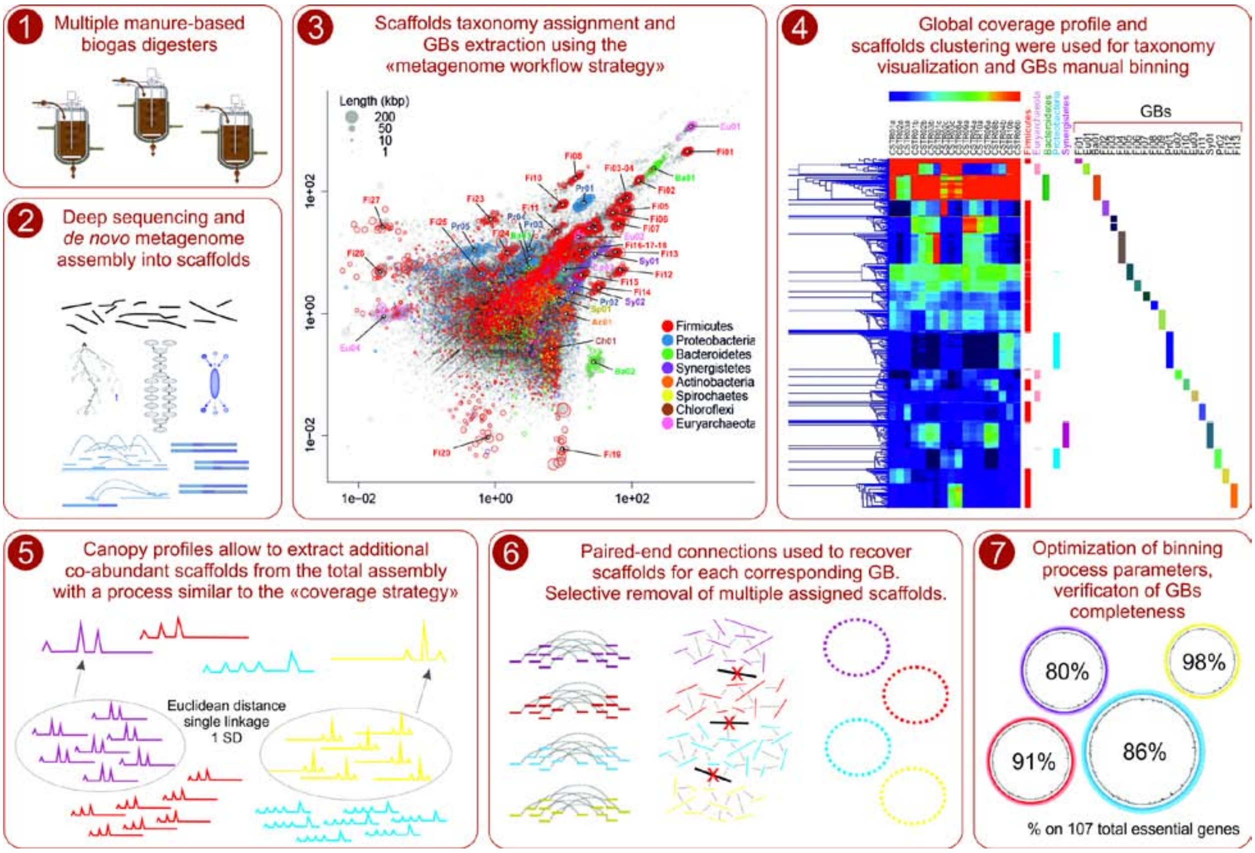


**Figure S3** Schematic representation of the binning strategy. (1) Genomic DNA was extracted from 8 manure-based digesters (18 samples in total). (2) Samples were shotgun-sequenced using Illumina (150 bp paired-end) and assembled using CLC Genomics workbench. (3) The resulting scaffolds were binned using the procedure proposed by Albertsen et al. [3]; at top left of the figure is reported the scaffold size legend, while at bottom right the taxonomic assignment legend with the eight more abundant Phyla. Circles in the graph are colored according to the taxonomic assignment and circle size is proportional to the scaffold length. In the x and y axes are reported respectively the scaffold coverage in two of the samples analyzed (CSTR01a and CSTR01b). In figure are reported some of the GBs identified using this procedure. (4) After scaffold coverage calculation in all the samples, the taxonomically assigned scaffolds were clustered considering the similarity of the coverage profile. At the right of the cluster image, the taxonomic assignment of the scaffolds and some of the GBs identified are reported. (5) After extraction of the taxonomically-assigned scaffolds from MeV clustering, a “canopy profile” was calculated and the scaffolds having similar coverage profiles were extracted from the entire scaffold list with a procedure similar to that proposed by Nielsen et al. [8]. (6) Scaffolds extracted for each GB and PE connections were analyzed and used to complete the GBs. (7) GBs completeness was estimated determining the presence in the scaffolds binned of 107 essential genes.

***Taxonomic assignment of the GBs.***

Taxonomic assignment of the GBs was performed with four different methods; results were then compared to extract the best possible one.

(1) The essential genes associated to each GB were checked by sequence similarity to the NR database using BLASTN, with e-value threshold 1e-5. Sequence similarity search of the 107 essential genes sequences was performed on the NR database and the taxonomic assignment of the best match was recovered. Sequence similarity of 95%, 85% and 75% or better was used for species, genus and phylum level taxonomical assignment [8], respectively. According to these thresholds, only 10 GBs were assigned to genus level (Additional File 3: Table S2) and no GBs were assigned at species level.

(2) The essential genes associated to each genome bin were checked by sequence similarity to the NR database using BLASTP, with e-value threshold 1e-5. Results obtained were analyzed and the most similar species are reported in Additional File 3: Table S2. Despite for all the GBs the most similar species was however distantly related, these results helped in the assignment of a putative functional role to the GBs.

(3) The scaffolds of each genome bin were analyzed using Phylopythia (http://phylopythias.cs.uni-duesseldorf.de/) with standard parameters [9]. For this specific analysis were considered only the results where more than 50% of the genome was assigned to the same taxonomy.

(4) Approximately 400 broadly conserved proteins were used to extract phylogenetic signal using Phylophlan [10] with standard parameters. Results were separated in “high”, “medium”, “low” and “incomplete” confidence (Additional File 3: Table S2). The same method provided the high-resolution microbial tree of life with taxonomic annotations (Figure 1).

Considering results from (3) and (4), GBs were manually taxonomically assigned according to the concordant result at the lower taxonomical level (Additional File 2: Table S2). Only 16 GBs were assigned to genus level, 21 at family level, 20 at class 47 at order and 2 at phylum level.

***Functional roles of the microbial species.***

The annotation (COG, KEGG, Pfam) of the genes assigned to the GBs can help to define the functional roles of the species identified. This analysis has been improved performing annotation of each GB using the SEED annotation subsystem [11]. Some categories relevant for functional characterization are reported in Additional File 8: Table S7. Hypergeometric analysis (Materials and methods) allowed the identification of the GBs with different functional roles. In Figure 3 only the GBs having *P* value lower than 0.05 in hypergeometric analysis and present in the top one eighth of each SEED/KEGG/COG functional category were reported (Additional Files 4-6: Tables S3-S5).

***COG analysis.***

Despite that COG classes describe very general functions, the analysis of the percentage of each COG class on each GB can highlight the functional roles of the species (Figure S4). These findings can be further confirmed and more specific functions can be identified by means of metabolic reconstruction (KEGG, SEED) [11, 12] and functional domains identification (Pfam) [13]. Considering the steps of the biogas production process, the most relevant COG classes to define functional roles are: (C) “energy production and conversion”, (E) “amino acid transport and metabolism”, (G) “carbohydrate transport and metabolism”, (H) “coenzyme metabolism” and (I) “lipid metabolism”. Class “C” has a crucial importance in the biogas microbial community, and GBs Eu05, Pr11, Sy01, Sy03 and Fi09 have a consistent fraction of their genes devoted to energy production (Figure S4). Of course, for *Methanothermobacter* sp. DTU051 (Eu05) and for other *Euryarchaeota*, this genome characteristic is due to their fundamental role in methanogenesis, while for other GBs this result is less obvious considering also their low abundance in the microbial community. Category “C” can be considered as strictly related to “G” (carbohydrate transport and metabolism), where the genomes having the higher number of genes are *Clostridia* (Fi15, Fi49) and Sy04. This result was confirmed by SEED subsystem where analysis of “carbohydrates” category revealed also that Fi09 is the genome with the highest number of subsystem feature counts in this category (Additional File 5: Table S4 and Figure S7). Category “H” (coenzyme metabolism) is dominated by the archaeal species, but obviously this result is determined by the high number of genes involved in the biosynthesis of the methanogenesis coenzymes. Interestingly, by analyzing the functional properties of the *Synergistetes* (Sy02, Sy03, Sy06), it was found that a high fraction of genes are related to amino acid transport and metabolism (category “E”) (Figure S4). This can be intriguingly linked to their suggested role in amino acids degradation, that resulted in the production of short-chain fatty acids and sulfate used by terminal degraders such as the methanogens and sulphate-reducing bacteria [14]. The presence of numerous features annotated as branched chain amino acids ABC-transporters in SEED supports this evidence at least for Sy01, Sy02, Sy03 and Sy06. Class “I” (lipid metabolism) is dominated by *Syntrophomonadaceae* (Fi07, Fi08, Fi09), Pr01 and *Alcaligenaceae* (Pr05, Pr06, Pr10).

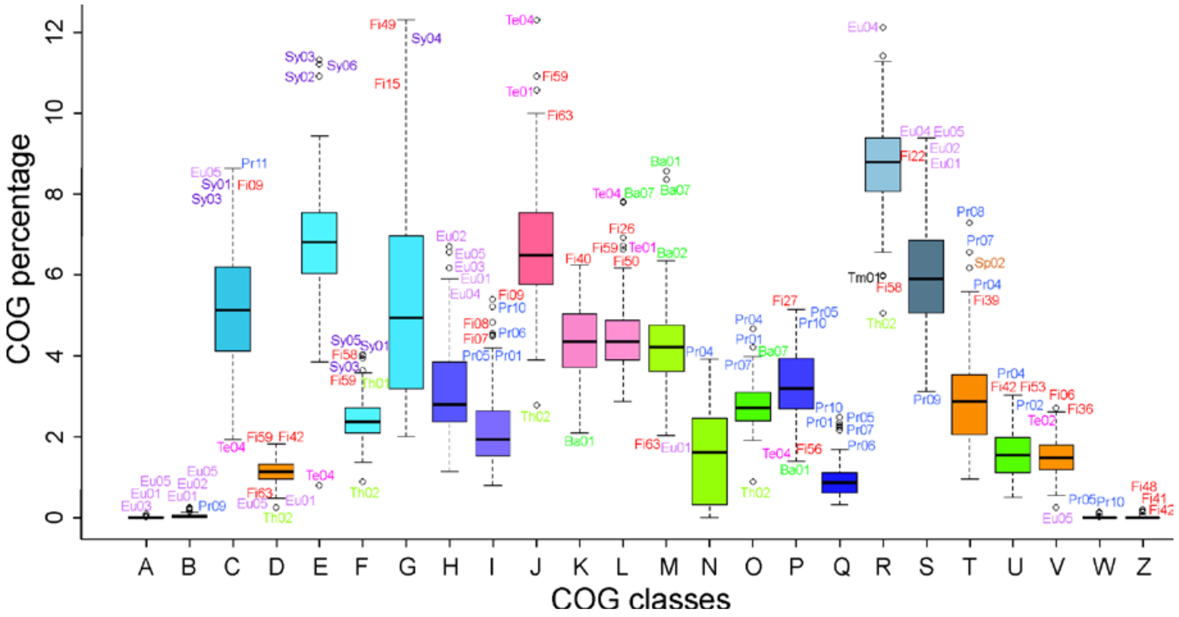


**Figure S4** Genes' fraction belonging to COG classes on each GB. For each functional COG class the percentage of genes identified for each GB is represented (y axes). Acronyms are reported for GBs having functional enrichments, colored by taxonomic assignment to Phylum level. Boxes are colored according to COG representation (ftp://ftp.ncbi.nih.gov/pub/COG/COG2014/static/lists/homeCOGs.html). The bottom and top of each box refers to the first and third quartiles, the band inside the box is the median, the whiskers are 1.5 fold the inner quartile range, dots represent the outliers.

***SEED analysis.***

SEED analysis was performed on each GB in order to tentatively assign them a functional role within the biogas community. In the SEED subsystem, the "subsystem category distribution" (feature counts) can be investigated at three levels; in the description we refer to the first two levels as "general" and "sub-categories". Results revealed that species with the higher number of genes in the general "carbohydrates" category (those in the first quartile) (Additional File 5: Table S4) can have 160 or more feature counts in this category and these are distributed in an heterogeneous way in the SEED sub-categories (i.e. some species possess a higher number of feature counts in some sub-categories and lower in others). This suggests specialized roles for groups of species in carbohydrate utilization and metabolism. The number of feature counts in the "sub categories" was used to generate the Network Representation of the Biogas Functional Organization (NRBFO) described in the main text (Figure 2). Briefly, this analysis converts the number of features in the SEED sub-categories (for each GB) in a network representation, and classifies the GBs considering their functional properties in "specialized" and "multifunctional". In the NRBFO the nodes represent the SEED classes and have a size proportional to the number of GBs present in the top one-eighth of the class. In some functional classes numerous GBs have no features and for this reason their size in the network is smaller.

As reported in the main text, most of the SEED categories for carbohydrate utilization (azure nodes in the network of Figure 2) are weakly connected suggesting specialized functions for the

bacterial species. On the contrary, genomes having high number of genes in more SEED categories are included in nodes connected by thick edges in the network (i.e. “fermentation” and “fatty acids”) which “share” 10 common genomes (Fi07, Fi08, Fi09, Fi12, Fi62, Fi68, Pr01, Pr02, Pr05, Pr10) (Additional File 5: Table S4). Among these, Fi07, Fi08 and Fi09 have numerous genes involved in “acetyl-CoA fermentation to butyrate”, “butanol biosynthesis” (pathways included in the general SEED category “fermentation”) and some are shared by the polyhydroxybutyrate metabolism” (a pathway included in the general SEED category “fermentation”). Diverse functions characterize the single species, for example only Fi07, Fi09, Fi12 and Pr05 have numerous genes in the “serine-glyoxylate cycle” suggesting that C1 assimilation involves butyryl-CoA and propionyl-CoA as intermediates (coming from polyxydroxybutyrate degradation).
GBs on each SEED sub-class were ranked considering their number of features. In this way it is possible to verify if the GBs present in the first 1/8$^{th}$ of each SEED category are also present in other SEED sub-categories. The GBs ranked in the first 1/8$^{th}$ of numerous categories have a “multifunctional purpose” in the microbial community. This characteristic is common in *Proteobacteria*, in fact considering the “multifunctional purpose” GBs, we found three members of the *Gammaproteobacteria* (Pr01, Pr02, Pr04), one of *Alcaligenaceae* (Pr10), but also two *Syntrophomonadaceae* (Fi07, Fi09), the remaining belong to some different groups (*Thermoanaerobacterales* Fi34, *Clostridiaceae* Fi40, *Peptococcaceae* Fi65). This is at a certain extent due to their slightly higher number of genes but this is not the only reason, since the range of genome sizes in the microbial community is quite small and 80% of the genomes have a reduced genome size ranging between 1.35 and 2.57 Mbp (Additional File 3: Table S2). Other microbial groups, for example *Clostridiaceae* comprise both members with a “multifunctional behavior” (i.e. Fi40, Fi48, Fi51, Fi30) and others more “specialized” (i.e. Fi23, Fi58, Fi50, Fi14). There is not a correlation between abundance in the microbial community and “behavior”, considering the ten most abundant genomes (in CSTR01a, 02a and 03a), Fi07 is “multifunctional” and Fi02, Fi06, Fi16 are more specialized.

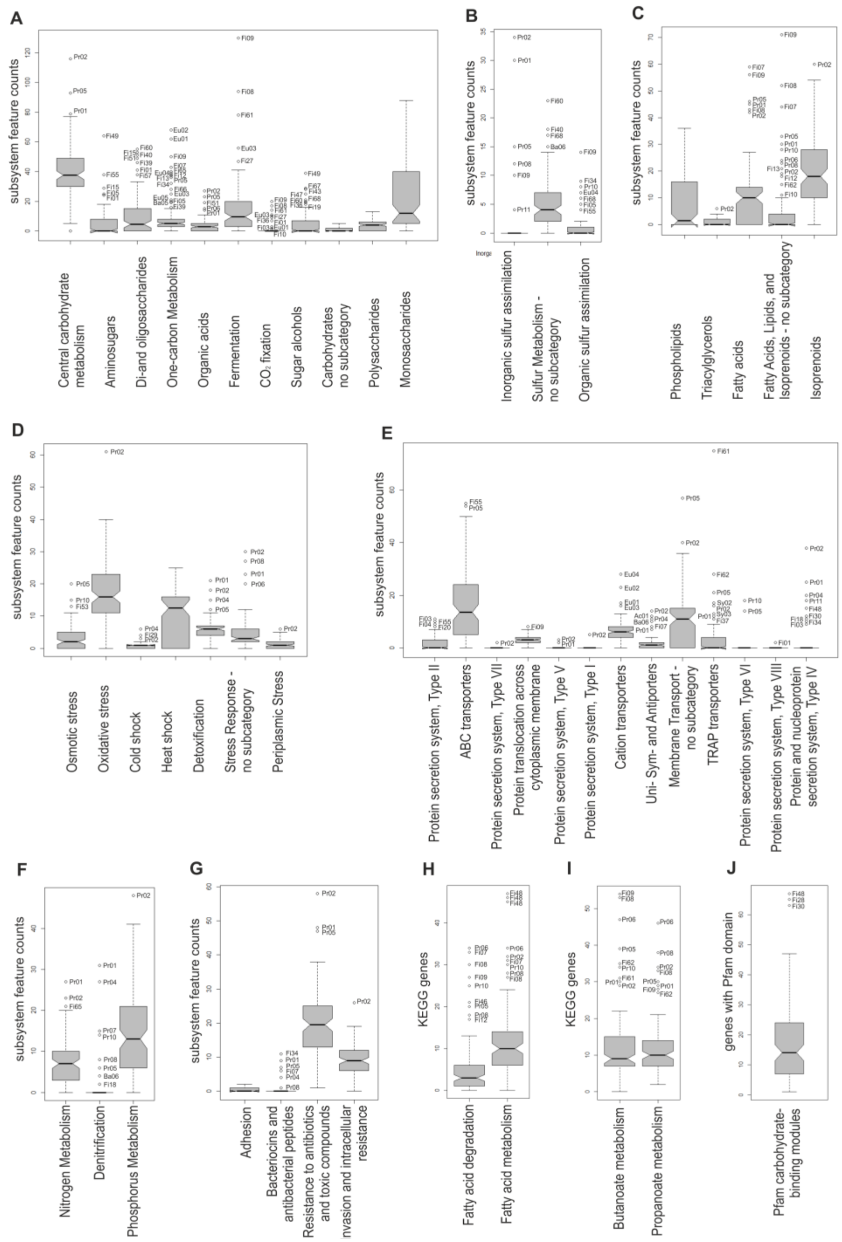


**Figure S7** Box plots obtained for some selected functional sub-classes. The number of subsystem features obtained for the 106 GBs on each of the SEED subcategories is reported in Additional File 5: Table S4. Box plot of some selected SEED sub-classes are reported in figure in order to show the distribution on the GBs of the number of subsystem features: (A) carbohydrates, (B) sulfur metabolism, (C) fatty acids, lipids, and isoprenoids, (D) stress response, (E) membrane transport, (F) nitrogen metabolism, phosphorus metabolism, (G) virulence, disease and defense. Box plot of some selected KEGG pathways are reported in (H) and (I), while in (J) the plot represents the number of genes having selected Pfam carbohydrate-binding modules. IDs of the GBs identified as outliers are reported.

***Number of genes for each KEGG class identified in the GBs.***

For each genome bin, the number of genes belonging to each KEGG category was calculated starting from Additional File 2: Table S1. Genes belonging to more than one KEGG class was considered once for each class. The matrix obtained with GBs in columns and KEGG classes in rows was uploaded to MeV software [5] and the gene number on each box was converted to a color scale ranging from white (zero genes) to red (50 genes). Red color in boxes with more than 50 genes is “saturated”. Columns (GBs) were clustered using Euclidean distance calculation (average linkage) and this procedure helped the identification of the more similar GBs in terms of gene copy number on KEGG categories. Some metabolic processes are particularly interesting for anaerobic digestion of organic matter like “propionate” and “butanoate” (butyrate) metabolism or “fatty acids degradation”. The identification of a high number of genes in specific KEGG categories for GBs, or the presence of “complete/nearly complete” KEGG pathways, assists the identification of the most interesting candidates for further investigations concerning their functional roles. Propionate and butanoate (butyrate) metabolisms were considered because they are central compounds in methanogenesis and they can be converted to acetate, $CO_2$ and $H_2$ and further used by methanogenic archaea for methane production. Lipids, together with carbohydrates and proteins were considered because they are the primary compounds used by the species belonging to the first layer of the microbial community that provide byproducts to the species of the lower layers.

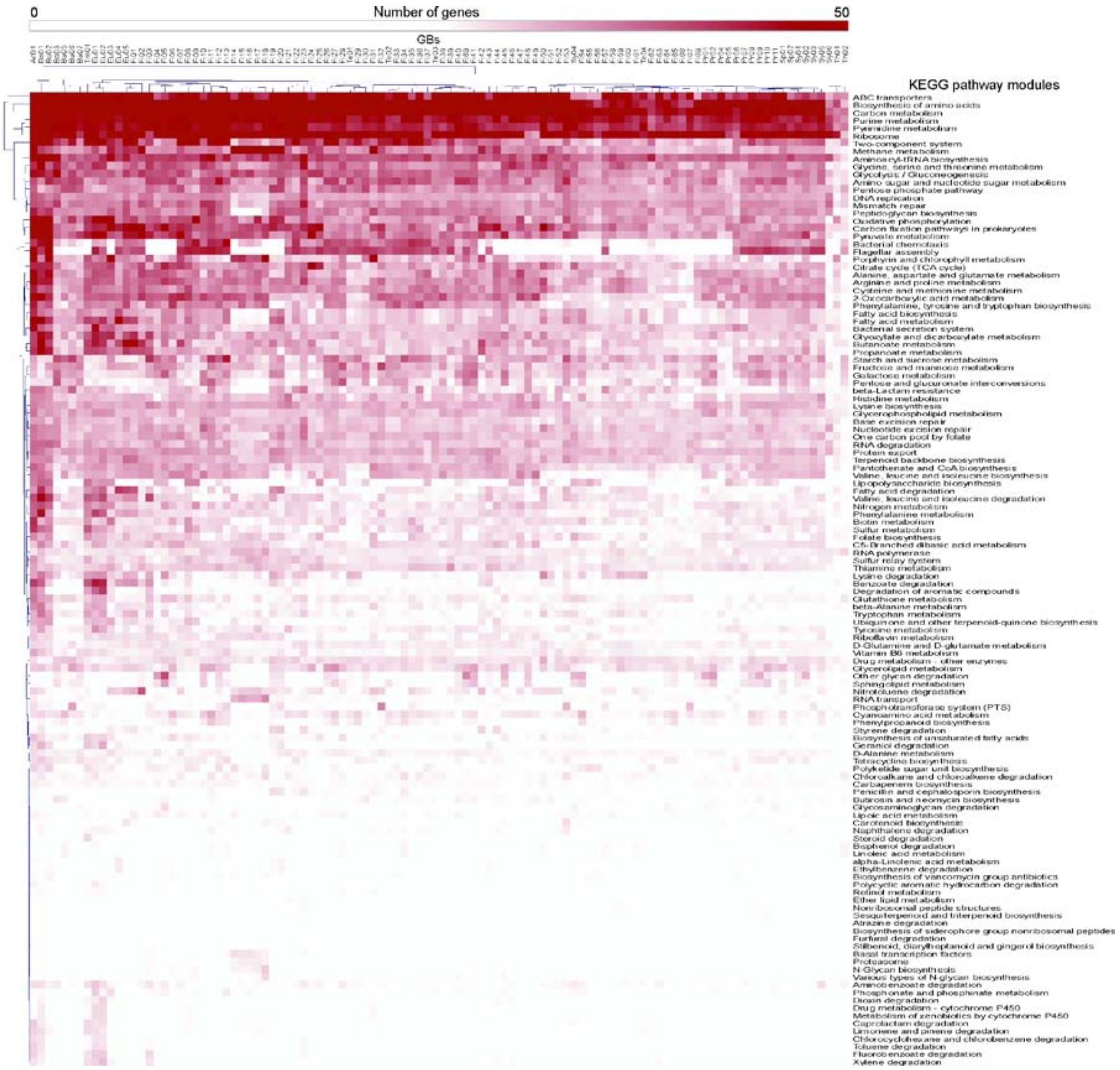


**Figure S5** Number of genes belonging to different KEGG pathway modules. Heat map representing the number of genes belonging to different KEGG pathways modules (rows) on each GB (columns). Color scale is reported in the upper part of the figure, GBs and KEGG pathways modules were clustered using Euclidean distance (average linkage) in order to have the most similar close to each other.

***Number of genes identified in the GBs for some selected KEGG pathways.***

Some selected KEGG pathways modules were investigated to identify genome bins having complete (or nearly complete) metabolic pathways. Acetate is the main precursor for methane in anaerobic digestion and two pathways for methane formation are known: aceticlastic methanogenesis carried out by *Methanosarcinaceae* and *Methanosaetaceae* and SAO performed by acetate-oxidizing bacteria in association with hydrogenotrophic methanogens (often *Methanomicrobiales* or *Methanobacteriales*) [15-17]. ABC transporters involved in carbohydrates and amino acid transport across the membrane were checked with the aim of identifying species particularly efficient in the utilization of selected primary compounds present in the organic matter supplied.

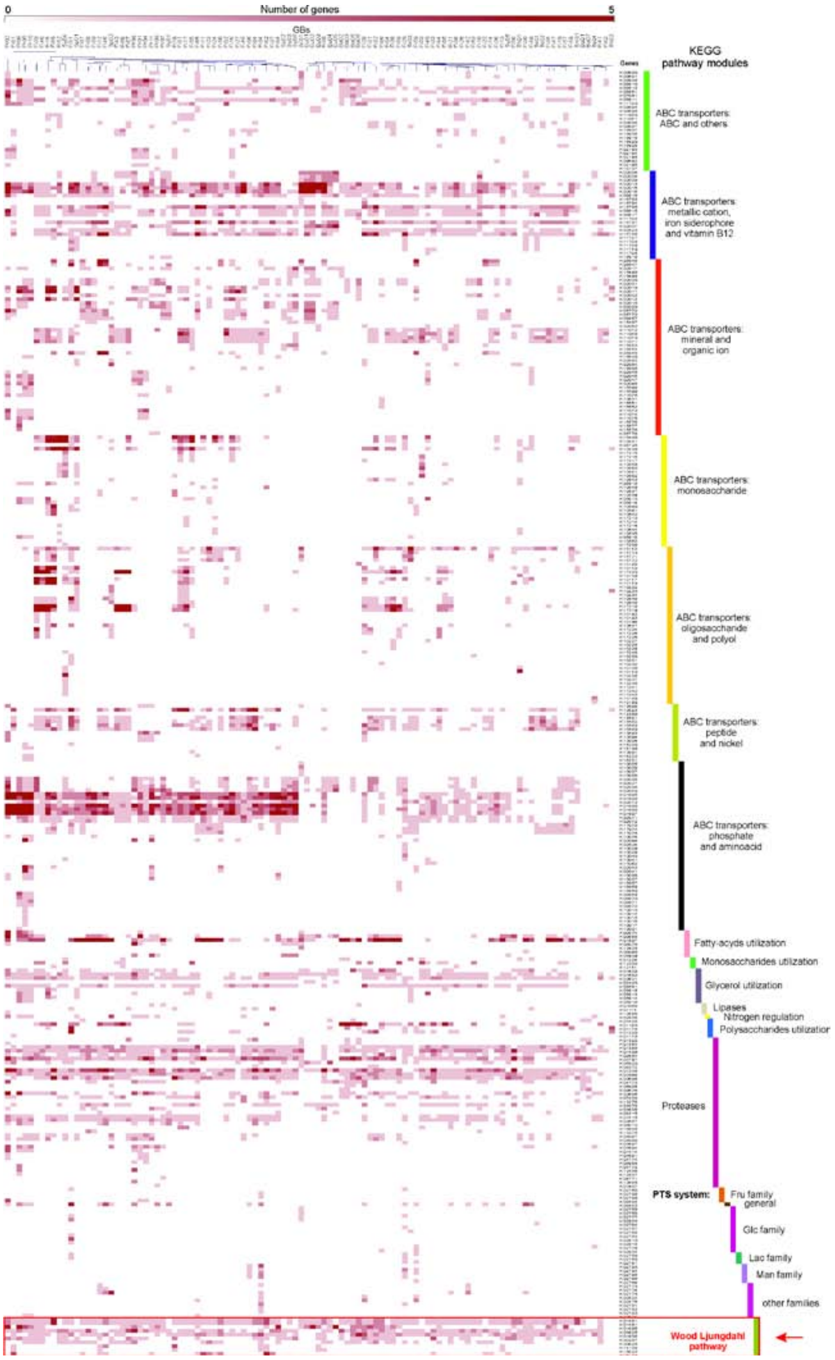


**Figure S6** Heat map of KEGG genes, selected pathways. Heat map representing the number of genes belonging to different KEGG IDs (rows) grouped considering functional categories

and reported for each GB (columns). KEGG IDs were selected and grouped in pathways considering those that are useful for functional assignment of the GBs to the functional roles in the biogas microbial community. Colors are correlated to the number of genes, the scale is reported on top of the figure. The Wood Ljungdahl pathway in the lower part of the figure is highlighted by a red box and an arrow.

***Methanogenic archaea.***

In previous microbial analyses performed using 16S rRNA or shotgun sequencing, it has been reported the presence of a large fraction of sequences assigned only to archaea with no additional taxonomic evidences [18]. This “archaeal dark matter” is of great interest but the small number of uncultured archaeal genomes in public databases made the analysis of the shotgun sequences through alignment on reference genomes very complex. In our study, assembly and binning led to the identification of an unknown *Euryarchaeota* (Eu03; *Euryarchaeota* sp. DTU008).

Comparative analysis of the SEED subsystem offers the great opportunity to have a first glimpse in the metabolic properties and phenotypic features of the Eu03 GB. In comparison to other methanogens of the biogas community it has a larger number of genome functions related to the “central carbohydrates metabolism”, the “amino acids and derivatives” (Alanine, serine, and glycine), the “fatty Acids, Lipids, and Isoprenoids” (similarly to the GB Eu02). On the contrary, it has a low number of genome functions in the “one-carbon metabolism” (the category comprising methanogenesis). The high number of molecular functions related to “lipids” are mainly involved in the archaeal membrane lipids biosynthesis, which are characterized by the (S)-2,3-di-O-geranylgeranylglyceryl phosphate (and variations) and also in biosynthesis of the isoprenoids side chains of the (respiratory) quinones.

The methane pathway was analyzed more in detail starting from the protein sequences of the 5 archaeal GBs (Figure 4). On each genome bin, the genes involved in methane pathway were identified using KEGG Automatic Annotation Server (KAAS) [19]**.** Organisms selected to perform KEGG analysis were chosen considering the species more similar to the GBs on the basis of previous phylogenetic analysis performed, and were: *Methanosarcina acetivorans* (mac), *Methanosarcina barkeri* (mba), *Methanosarcina mazei* Go1 (mma), *Methanococcoides burtonii* (mbu), *Methanoculleus marisnigri* (mem), *Methanothermobacter thermautotrophicus* (mth), *Methanothermobacter marburgensis* (mmg), *Pyrococcus horikoshii* (pho), *Pyrococcus abyssi* (pab), *Pyrococcus furiosus* DSM 3638 (pfu), *Thermoplasma acidophilum* (tac), *Thermoplasma volcanium* (tvo). For all the archaeal GBs identified in this study, the methane pathway shows a high level of completeness and this underlines the high quality level of the genome assembly and binning procedure. The reference KEGG methane metabolism pathway, comprising the biosynthesis of the coenzyme F420 (which is absent in *Ca. M. thermitum* and in *Euryarchaeota* sp. DTU008) and the trimethylamine metabolism (which is present in *Methanosarcinales* and in *Euryarchaeota* sp. DTU008) are reported in Figure 4.

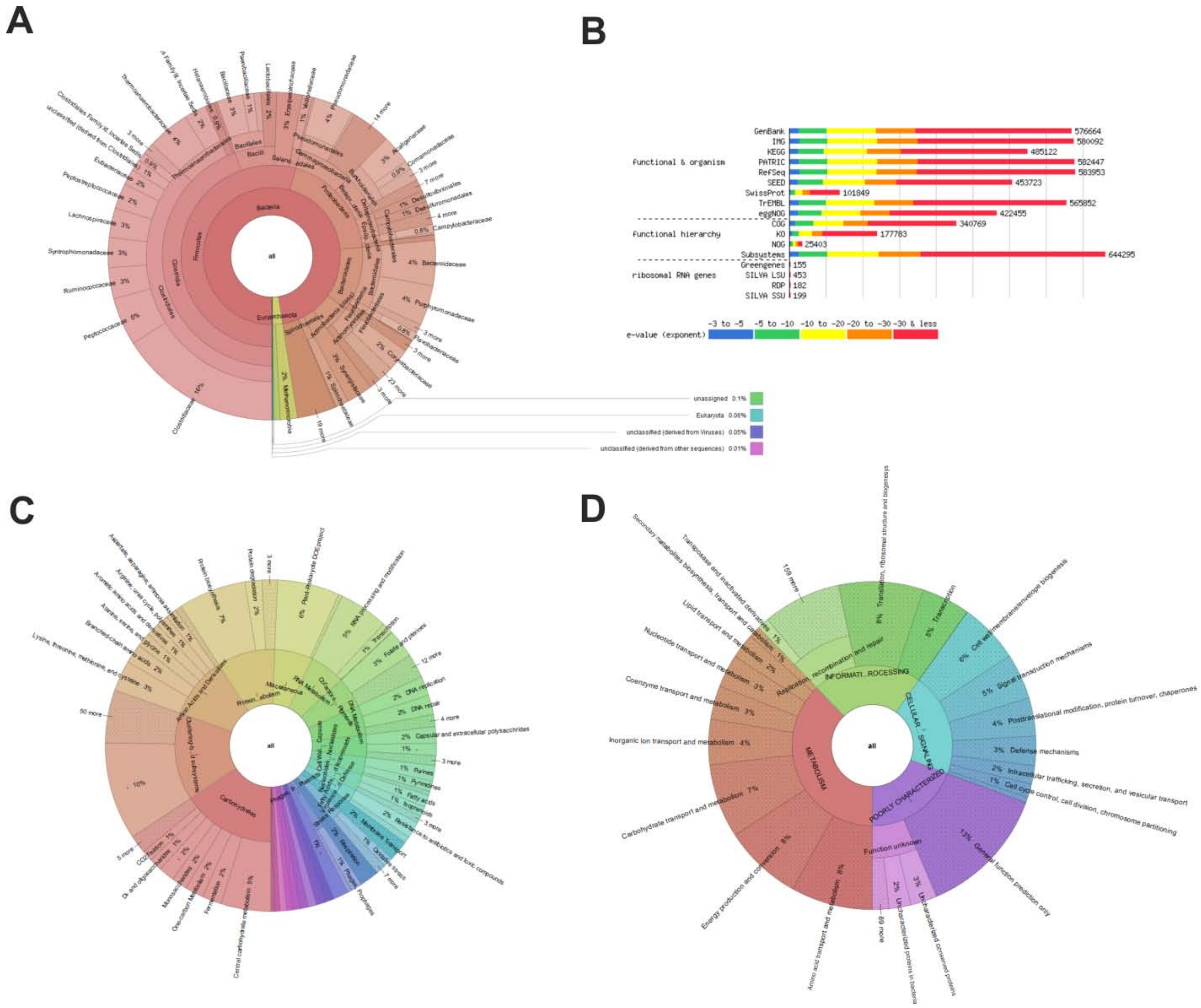


**Figure S8** Overview of the taxonomic and functional results obtained for the global metagenome assembly using MG-RAST server. (A) Taxonomic assignment of the scaffolds performed considering the RefSeq database. As expected the three most abundant groups identified from taxonomic assignment of the GBs (*Firmicutes*, *Proteobacteria* and *Bacteroidetes*) were confirmed, while a lower number of scaffolds were assigned to other groups like *Actinobacteria*, *Spirochaetes* and *Euryarchaeota*. (B) Results of the similarity search performed both for protein-encoding and for rRNA genes on different databases. (C) (D) Functional hierarchy obtained for SEED and COG.

## Additional references


1. Langmead B, Salzberg SL: Fast gapped-read alignment with Bowtie 2. Nature methods. 2012;9(4)**:**357-59.
2. Quinlan AR, Hall IM: BEDTools: a flexible suite of utilities for comparing genomic features. Bioinformatics. 2010;26(6)**:**841-42.
3. Albertsen M, Hugenholtz P, Skarshewski A, Nielsen KL, Tyson GW, Nielsen PH: Genome sequences of rare, uncultured bacteria obtained by differential coverage binning of multiple metagenomes. Nature biotechnology. 2013;31(6)**:**533-38.
4. Huson DH, Auch AF, Qi J, Schuster SC: MEGAN analysis of metagenomic data. Genome research. 2007;17(3)**:**377-86.
5. Saeed A, Sharov V, White J, Li J, Liang W, Bhagabati N, Braisted J, Klapa M, Currier T, Thiagarajan M: TM4: a free, open-source system for microarray data management and analysis. Biotechniques. 2003;34(2)**:**374.
6. Parks DH, Imelfort M, Skennerton CT, Hugenholtz P, Tyson GW: CheckM: assessing the quality of microbial genomes recovered from isolates, single cells, and metagenomes. Genome research 2015;25(7)**:**1043-55.
7. Chain P, Grafham D, Fulton R, Fitzgerald M, Hostetler J, Muzny D, Ali J, Birren B, Bruce D, Buhay C: Genome project standards in a new era of sequencing. Science (New York, NY). 2009;326(5950).
8. Nielsen HB, Almeida M, Juncker AS, Rasmussen S, Li J, Sunagawa S, Plichta DR, Gautier L, Pedersen AG, Le Chatelier E: Identification and assembly of genomes and genetic elements in complex metagenomic samples without using reference genomes. Nature biotechnology. 2014;32(8)**:**822-28.
9. Patil KR, Haider P, Pope PB, Turnbaugh PJ, Morrison M, Scheffer T, McHardy AC: Taxonomic metagenome sequence assignment with structured output models. Nature methods. 2011;8(3)**:**191-92.
10. Segata N, Börnigen D, Morgan XC, Huttenhower C: PhyloPhlAn is a new method for improved phylogenetic and taxonomic placement of microbes. Nature communications. 2013;4.
11. Overbeek R, Olson R, Pusch GD, Olsen GJ, Davis JJ, Disz T, Edwards RA, Gerdes S, Parrello B, Shukla M: The SEED and the Rapid Annotation of microbial genomes using Subsystems Technology (RAST). Nucleic acids research. 2014;42(D1)**:**D206-D14.
12. Kanehisa M, Goto S, Sato Y, Kawashima M, Furumichi M, Tanabe M: Data, information, knowledge and principle: back to metabolism in KEGG. Nucleic acids research. 2014;42(D1)**:**D199-D205.
13. Finn RD, Bateman A, Clements J, Coggill P, Eberhardt RY, Eddy SR, Heger A, Hetherington K, Holm L, Mistry J: Pfam: the protein families database. Nucleic acids research. 2013gkt1223.
14. Vartoukian SR, Palmer RM, Wade WG: The division "*Synergistes*". Anaerobe. 2007;13(3)**:**99-106.
15. Hattori S, Kamagata Y, Hanada S, Shoun H: *Thermacetogenium phaeum* gen. nov., sp. nov., a strictly anaerobic, thermophilic, syntrophic acetate-oxidizing bacterium. International Journal of Systematic and Evolutionary Microbiology. 2000;50(4)**:**1601-09.
16. Petersen SP, Ahring BK: Acetate oxidation in a thermophilic anaerobic sewage-sludge digestor: the importance of non-aceticlastic methanogenesis from acetate. FEMS microbiology ecology. 1991;9(2)**:**149-57.
17. Schnürer A, Svensson BH, Schink B: Enzyme activities in and energetics of acetate metabolism by the mesophilic syntrophically acetate-oxidizing anaerobe *Clostridium ultunense*. FEMS microbiology letters. 1997;154(2)**:**331-36.
18. Wirth R, Kovács E, Maróti G, Bagi Z, Rákhely G, Kovács KL: Characterization of a biogas-producing microbial community by short-read next generation DNA sequencing. Biotechnol Biofuels. 2012;5(1)**:**41.
19. Moriya Y, Itoh M, Okuda S, Yoshizawa AC, Kanehisa M: KAAS: an automatic genome annotation and pathway reconstruction server. Nucleic acids research. 2007;35(suppl 2)**:**W182-W85.
